# Highly sampled tetranucleotide and tetraloop motifs enable evaluation of common RNA force fields

**DOI:** 10.1261/rna.051102.115

**Published:** 2015-09

**Authors:** Christina Bergonzo, Niel M. Henriksen, Daniel R. Roe, Thomas E. Cheatham

**Affiliations:** Department of Medicinal Chemistry, College of Pharmacy, L.S. Skaggs Pharmacy Research Institute, University of Utah, Salt Lake City, Utah 84112, USA

**Keywords:** enhanced sampling, replica exchange, molecular dynamics, RNA, AMBER, CHARMM, force fields

## Abstract

Recent modifications and improvements to standard nucleic acid force fields have attempted to fix problems and issues that have been observed as longer timescale simulations have become routine. Although previous work has shown the ability to fold the UUCG stem–loop structure, until now no group has attempted to quantify the performance of current force fields using highly converged structural populations of the tetraloop conformational ensemble. In this study, we report the use of multiple independent sets of multidimensional replica exchange molecular dynamics (M-REMD) simulations with different initial conditions to generate well-converged conformational ensembles for the tetranucleotides r(GACC) and r(CCCC), as well as the larger UUCG tetraloop motif. By generating what is to our knowledge the most complete RNA structure ensembles reported to date for these systems, we remove the coupling between force field errors and errors due to incomplete sampling, providing a comprehensive comparison between current top-performing MD force fields for RNA. Of the RNA force fields tested in this study, none demonstrate the ability to correctly identify the most thermodynamically stable structure for all three systems. We discuss the deficiencies present in each potential function and suggest areas where improvements can be made. The results imply that although “short” (nsec-μsec timescale) simulations may stay close to their respective experimental structures and may well reproduce experimental observables, inevitably the current force fields will populate alternative incorrect structures that are more stable than those observed via experiment.

## INTRODUCTION

Molecular dynamics simulations of nucleic acids are important for understanding motions over multiple timescales, which may be inaccessible by experimental methods, and also for helping to clarify obscure experimental results (Cheatham and Case 2013). In particular, RNA performs many different active roles within cells, making it important to accurately model structure and dynamics over multiple timescales (Meister 2011). This means that the accuracy of the potential function, or force field, is of the utmost importance when performing simulations of these highly charged and highly flexible polymers.

Historically, in both protein and nucleic acid force field development, a significant difficulty in evaluating force fields has been the inability to clearly decouple errors in the potential function from errors caused by incomplete sampling of the conformational ensemble (Pérez et al. 2012). A step in the right direction has been the application of enhanced sampling methods which have been used to identify problems and validate updates to force field parameter sets (Mlýnský et al. 2010; Chen and García 2013; Nguyen et al. 2014). Widely applied among enhanced sampling methodologies are replica exchange molecular dynamics (REMD) simulations, which have become a common way to evaluate conformational ensembles of RNA (Sorin et al. 2002; Vaiana and Sanbonmatsu 2009; Chen and García 2013; Henriksen et al. 2013; Kührová et al. 2013; Bergonzo et al. 2014; Roe et al. 2014). In REMD, several independent simulations are run at different temperatures or Hamiltonians (referred to as T-REMD and H-REMD, respectively), and exchanges are attempted between them at specific intervals (Hansmann 1997; Sugita and Okamoto 1999). During these simulations the lower temperature replicas benefit from access to structures sampled at higher temperatures, where enthalpic barriers are more easily crossed, and the aggregate sampling for the entire simulation is improved. For example, REMD simulations can be used to explore additional conformational space during protein and nucleic acid folding simulations (Simmerling et al. 2002; Sorin et al. 2002; Vaiana and Sanbonmatsu 2009; Kührová et al. 2013). Access to GPU-enabled code and other enhanced sampling methods such as accelerated MD have enabled these types of simulations to routinely access the microsecond time scale (Hamelberg et al. 2004; Salomon-Ferrer et al. 2013; Roe et al. 2014).

RNA structure is complex and involves a subtle balance between charge interactions, hydrogen bonding, stacking contacts, backbone conformational flexibility, sugar puckers, and glycosidic torsions, all adding significant difficulty to potential function development. Though modest success has been seen with regard to reevaluating experimental data and qualitatively describing dynamics, force fields which can fold RNA, i.e., predict their tertiary or secondary structure, are lacking compared with those used in protein simulations (Laing and Schlick 2011). The benchmarks of robust RNA force fields have traditionally been folding tetraloops, a structure characterized by an A-form helix stem capped by a 4-nt loop. These tetraloop motifs are prevalent in biology (Uhlenbeck 1990; Bevilacqua and Blose 2008). The UNCG and GNRA tetraloop motifs (where N is any nucleotide and R is a purine) compose >70% of tetraloops in the 16S ribosomal RNA subunit (Woese et al. 1990). Though there is a significant amount of structural data which points to a consensus structure (Ennifar et al. 2000; Nozinovic et al. 2010), the UUCG tetraloop in particular is poorly described by current force fields as evidenced by longer simulations, which show further drift from the experimental energy minimum (Banáš et al. 2010).

Routine access to longer simulation times has shown the breakdown of some popular force fields for nucleic acids. Modifications to the AMBER ff99 (Cheatham et al. 1999; Wang et al. 2000) nucleic acid force field corrected overpopulation of the α/γ *gauche+*, *trans* backbone conformation (Pérez et al. 2007b). These populations were seen in long MD simulations on the order of tens of nanoseconds, and the dihedral corrections were validated using longer MD simulations up to 1 μsec in length (Pérez et al. 2007a). RNA-specific modifications were also made to the glycosidic χ torsion after simulations of the hairpin ribozyme transitioned to unnatural ladder-like structures (Mlýnský et al. 2010; Zgarbová et al. 2011). Alternate χ torsion modifications were made around the same time, motivated by poor agreement with experimental NMR data (Yildirim et al. 2012). These last sets of modifications were validated over 500-nsec replica exchange simulations and long MD simulations on the order of microseconds, respectively.

Though modifications to dihedral parameters have dominated force field corrections, recent parameters have addressed the various problems with the nonbonded components (Chen and García 2013). Reparameterization of the van der Waals (vdW) radii to more closely match experimental values, as well as the modification of off-diagonal Lennard-Jones (LJ) terms which address the imbalance in solute–solvent interactions was performed based on the observations seen in high-level QM calculations (Chen and García 2013). With these parameters it is possible to fold the stem–loop motif of tetraloops via replica exchange simulations on the order of 500 nsec per replica.

**FIGURE 1.**
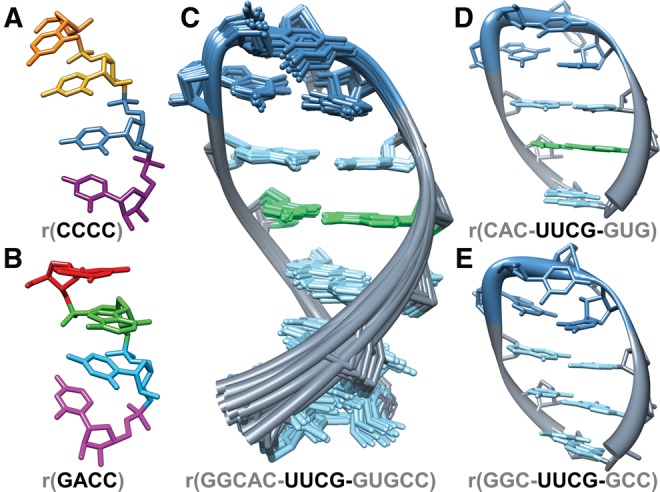
Images of RNA systems simulated in this study include (*A*) the r(CCCC) tetranucleotide, shown in an A-form conformation with bases colored by residue number, (*B*) the r(GACC) tetranucleotide, shown in the NMR major conformation with bases colored by residue number, (*C*) the r(GGCAC-UUCG-GUGCC) 2KOC NMR ensemble, where the A:U base pair is shown in green, G:C base pairs are shown in light blue, and the UUCG loop sequence is shown in dark blue, (*D*) r(CAC-UUCG-GUG), a truncated version of structure 1 from the 2KOC NMR ensemble, used as a starting structure for M-REMD simulations, and (*E*) The UUCG tetraloop with alternate stem sequence, used as a starting structure for Anton simulations. The G:C base pairs are shown in light blue and the UUCG loop sequence in dark blue. We note that an alternative stem structure was used in the Anton simulations (in the 2010–2011 timeframe) to be consistent with our earlier tetraloop investigations. In this earlier study, representatives of all tetraloop structures found in the PDB were grafted onto a common stem structure; these relatively short MD simulations with even more poorly performing force fields were never published.

Though these more recent force fields can reproducibly fold tetraloop structures, the simulations admittedly remain unconverged (Chen and García 2013; Kührová et al. 2013). The unconverged nature of these simulations critically limits the evaluation of force field modifications, and calls into question the appropriateness of any particular force field for simulating alternate systems. In other words, the robustness of the force field remains uncertain. The goals of this study are to remove the obstacle of sampling from force field assessment by using multidimensional replica exchange molecular dynamics (M-REMD) to sample configurational space to a higher degree of convergence. Although true and complete convergence of a conformational ensemble is elusive and difficult to prove or fully define, we show reproducible results, determined from cluster populations and principle modes of motion, from multiple sets of independent simulations with different initial conditions and even application of different enhanced sampling methodologies, for example, comparing accelerated MD to H-REMD (Bergonzo et al. 2014; Roe et al. 2014). In this study, we compare the resulting ensembles of the top RNA force fields currently in use, and described in detail in Table 1. We show highly converged structure ensembles for the UUCG tetraloop, as well as the r(CCCC) and r(GACC) tetranucleotides (structures shown in Fig. 1) for a variety of force fields. We note the deficiencies present in each potential function, and highlight areas to address for future improvement.

**TABLE 1.**
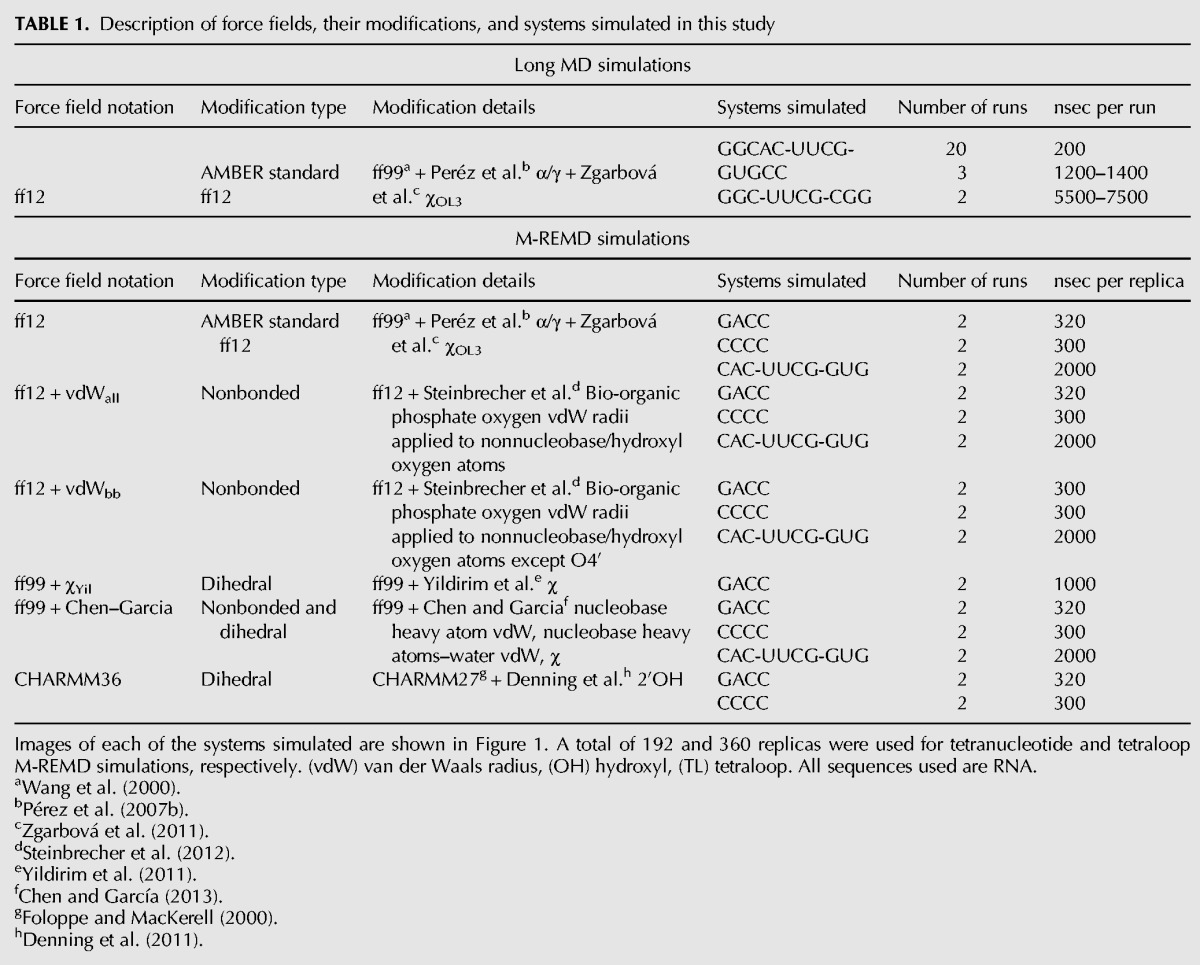
Description of force fields, their modifications, and systems simulated in this study

## RESULTS

### Instability in standard MD simulations of the UUCG tetraloop on multiple timescales

**FIGURE 2.**
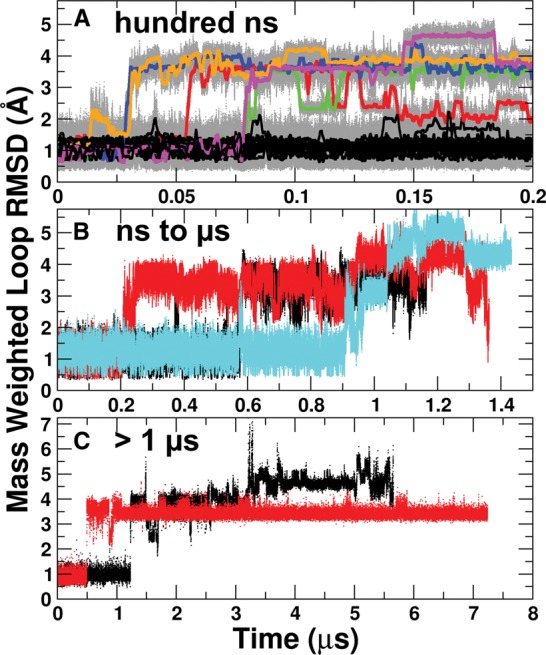
Mass-weighted loop RMSD (UUCG loop and closing base pair are included) versus time. (*A*) Twenty 200-nsec simulations of UUCG, starting from each of the 20 members of the NMR ensemble. RMSDs are shown with all data in gray, and 1-nsec running averages of each simulation in black (if they maintain native contacts) or in color (if native loop contacts are lost). (*B*) Three >1-μsec simulations of UUCG, performed on a combination of CPUs and GPUs, starting from the first three members of the NMR ensemble. (*C*) Two >5-μsec simulations performed on Anton. Increases in the RMSD can be attributed to a loss of loop structure.

A large set of independent MD simulations of the UUCG tetraloop with sequence r(GGCAC-UUCG-GUGCC) were performed using AMBER ff12 on GPUs (an ensemble of 20 independent runs of 200 nsec each), CPUs and GPUs (three independent runs of 1.2–1.4 µsec each), and also with sequence r(GGC-UUCG-GCC) on the Anton machine at PSC (two independent runs of 5.5 and 7.4 µsec each). Representations of the UUCG tetraloop systems studied in these MD simulations are shown in Figure 1C,E. The persistence of the NMR-refined RNA conformation varies within each of the twenty 200-nsec simulations. Five of the 20 simulations display a major distortion of the UUCG tetraloop that precedes a large jump in the loop RMSD value (Fig. 2). We refer to these unstable simulations as unstable5 and the remaining as stable15. To illustrate the changes in the loop conformation for both the stable15 and unstable5 subsets, we show the overlap of frames from each simulation on the NMR structure (Model 1) in Supplemental Figure 1. For the unstable5 simulations, the primary change in the tetraloop appears to be the loss of hydrogen bonding between U_L1_ and G_L4_ followed by the extrusion of G_L4_ into the bulk solvent. In one case (red line in Fig. 2), hydrogen bonds reform between U_L1_ and G_L4_, however, the χ orientation of the G_L4_ base adopts an *anti* value rather than the original *syn*. In none of the unstable5 simulations does reformation of the canonical UUCG conformation occur.

Although the UUCG tetraloop contacts are largely maintained in the stable15 simulations, the simulation results do deviate from the NMR ensemble (as well as violating the distance/torsion restraint bounds) in subtle but consistent ways. This deviation is evident in the backbone near residues C_L3_, G_L4_, and G_L4+1_ of the loop-capping base pair. Two torsions, in particular, appear to be problematic: the G_L4_ ε and the G_L4+1_ β of the loop-capping CG base pair. Although the G_L4_ ε adopts the *trans* region based on the NOEs measured in the NMR ensemble, it consistently adopts a high-*anti* value during simulation. The capping G_L4+1_ β is not restrained in the NMR ensemble but β torsions are nearly always *trans* in RNA structures. In the case of the stable15 simulations, β adopts an unexpected value near ∼80°. It is worth noting that the dinucleotide conformation from G_L4_ to capping G_L4+1_ in the configuration sampled in the stable15 simulations does not belong to any of the populated RNA dinucleotide conformational suites identified by Richardson et al. (2008), and when the original NMR models are checked using the *Suitename* program, the suite receives the “triaged” assignment, indicating it contains at least a single backbone dihedral angle outlier.

The mass-weighted loop RMSD for two additional sets of simulations are shown in Figure 2B,C: three simulations run on both GPUs and CPUs, each longer than 1 µsec, and two simulations run on the Anton supercomputer, each longer than 5 µsec. These simulations show the UUCG loop conformation and dynamics on longer timescales and not only help to assess and validate the force field performance, but show that results obtained with the CPU and GPU versions of the PMEMD code behave similarly and also that similar results are seen with the specialized Anton supercomputer (Shaw et al. 2008). Each of the simulations experience UUCG tetraloop disruption after which the NMR conformation never reforms on the timescale of the simulations. In all cases, residue G_L4_ appears to be responsible for the disruption and always flips out into the bulk solvent. As was the case for the 200-nsec simulations, the backbone torsions between residue G_L4_ and the capping G deviate from those present in the NMR model. The Anton simulation that maintains a 3 Å RMSD (Fig. 2C, red) from the NMR structure has the G_L4_ residue completely flipped from *syn* to *anti*, and has reformed some of the characteristic hydrogen bonds in the wobble G–U base pair. Additionally, the other Anton simulation (Fig. 2C, black) completely unfolds after 3 μsec. The unfolding event is preceded by disruption of the canonical UUCG conformation at 1.2 μsec, after which the loop samples a variety of stacking dominated configurations until finally a rearrangement of the Watson–Crick pairing in the stem region leads to complete unfolding. As the structure is disrupted in all cases on the longer MD simulation timescales, these results are not necessarily sufficient to validate the CPU versus GPU versus Anton runs, despite the similar behavior. However, our group has recently demonstrated the similarity of results from simulations performed on GPUs, CPUs, and Anton via MD simulations of DNA that show convergence of the conformational ensemble of the internal part of the DNA helix on the 1–5-μsec timescale (Galindo-Murillo et al. 2014a,b).

Taken together, these MD simulations of the UUCG tetraloop show greater and persistent loss of tetraloop structure as simulation timescales increase. However, more sampling is needed to converge the tetraloop structure in order to quantify and to better identify problems with the force fields. To compare the converged structure ensembles across many force fields, we extend the sampling by using M-REMD methods. In addition, we performed M-REMD simulations using two RNA tetranucleotides, r(GACC) and r(CCCC), for which more simulation data (and ultimately better convergence) can be obtained due to their smaller size. NMR data are also available for these tetranucleotides, which allows validation of the force field's resulting ensembles (Yildirim et al. 2012; Tubbs et al. 2013).

### Force fields predict a diverse set of r(GACC) tetranucleotide structures

**FIGURE 3.**
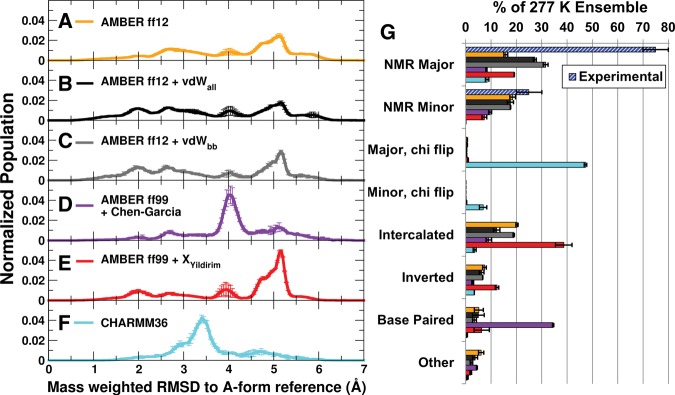
(*Left*) M-REMD RMSD to A-form Reference for r(GACC). (*A*) ff12, (*B*) ff12 + vdW_all_, (*C*) ff12 + vdW_bb_, (*D*) ff99 + Chen–Garcia, (*E*) ff99 + χ_Yil_, and (*F*) C36 force fields. The averages between two runs per force field are shown, with error bars shown as the standard deviation. RMSDs corresponding to NMR major and minor structures are ∼2.0 Å and 2.6 Å, respectively. (*G*) Populations of major conformation types seen in cluster analysis of each M-REMD simulation. Bars indicate the average and standard deviation between two independent runs and are colored by force field to match the RMSD histograms. Experimental values are shown on the table in a blue striped pattern. Representative structures and classifications (including discussion of the conformations classified as “Other”) are shown in Supplemental Table 1 and Supplemental Figure 2.

Given the difficulties with sampling tetraloop structures in straight MD simulations with a wide variety of force fields over a number of years by our group, we moved to smaller tetranucleotide systems in order to enable more thorough sampling of conformational space. With highly optimized AMBER MD engines on GPUs (Case et al. 2012; Götz et al. 2012; Salomon-Ferrer et al. 2013) and with access to large-scale GPU resources such as NICS Keeneland and NCSA Blue Waters, it is now becoming routine to more fully sample (and ideally “converge”) ensembles of these smaller, but still conformationally diverse, systems (Bergonzo et al. 2014). Well-sampled distributions of GACC are shown as histograms of the 277 K replica's mass-weighted RMSD from a canonical A-form tetranucleotide in Figure 3. Effective agreement between independent sets of M-REMD simulations with different initial conditions is shown in the Supplemental Table 1. As is evident from the data, the different force fields sample each conformation in the ensemble with a different probability, however, the variance between independent runs with the “same” force field is rather small, typically <2% or effectively less than kT (see Supplemental Table 1). Given the agreement between independent sets of simulations with different initial conditions, including agreement in principal modes of motion sampled (even with the application of different enhanced sampling methodologies) we believe we have demonstrated reproducible and equivalent results. Although this does not “prove” convergence of the conformation distribution since it is possible we have not sampled sufficiently to find elusive or hidden conformations, we think we are approaching convergence in the conformational distributions of tetranucleotides.

In Figure 3A, peaks in the RMSD to A-form reference at 2.0 Å and 2.6 Å correspond to the experimentally determined NMR major and NMR minor structures, respectively. It is apparent that for most of the force fields these structures are *not* the most highly populated members of the ensemble as experiment predicts they should be. As discussed in previous work, the alternative structures that are populated are not consistent with the NMR data (Henriksen et al. 2013). We also investigated the ff99 + χ_Yil_ force field modifications which were specifically developed to correct the χ dihedral by fitting to high-level QM potentials, and were shown to reproduce the correct proportion of GACC NMR major and minor structures by way of several MD simulations: fifteen ∼50 nsec in length, and one ∼1.9 µsec in length (Yildirim et al. 2012). However, Yildirim and colleagues also reported that they observed another non-A-form structure with significant population in their longer MD simulation. The results from our independent M-REMD simulations using these parameters (192 replicas, ∼1 µsec per replica) do in fact show that intercalated structures that are inconsistent with the NMR data are the main representatives of the ensemble, accounting for ∼40% of structures (including a conformation consistent with the non-A-form one reported by Yildirim et al. 2011). While the overall populations of NMR major and NMR minor from the ff99 + χ_Yil_ simulations do not reproduce the experimentally determined populations, the ratio of NMR major to NMR minor is roughly 2.5:1, which is consistent with the relative populations of these two structures from the experimental ensemble; we note that no other force field tested was able to do this.

The current AMBER force field for RNA, referred to here as ff12 (though there is no difference from ff14) also incorrectly predicts the ensemble. Discussed at length in our previous paper, though NMR major and NMR minor populations are detected, the most populated structures are inverted and intercalated. We hypothesized that increasing the vdW radii of the phosphate oxygen atom types, following the modifications by Steinbrecher et al. (2012), would destabilize the more compact structures, leading to an increase in extended structures. These force fields are referred to as ff12 + vdW_all_ (all nonnucleobase/nonhydroxyl oxygen atoms modified) and ff12 + vdW_bb_ (all nonnucleobase/nonhydroxyl oxygen atoms except O4′ modified). In simulations with both of these modified force fields, the NMR major becomes the most populated structure. The ratio of NMR major to NMR minor is higher in ff12 + vdW_bb_ at 1.8:1 than in ff12 + vdW_all_ at 1.6:1. These modified force fields correctly predict the NMR major and NMR minor structures as the top two clusters, respectively. Even so, there are significant populations of alternate structures (specifically intercalated structures), which have a total population ∼10% in ff12 + vdW_all_ and 15% in ff12 + vdW_bb_. These populations are large enough such that if they occurred in experiments, one might expect them to be detectable by NMR. The rest of the sampled structures are each <10% populated. Structures with <10% population are likely not populated enough to yield signals which are distinguishable from instrumental noise in the solution NMR to which we are comparing, so it remains unknown if these are reasonable structures which exist in nature or if they are solely the result of force field bias. Taken together, the clustering results indicate that increasing the vdW radius of nonnucleobase oxygen atom types does reduce the amount of intercalated structure found (vdW_all_ has an additional O4′ radius enlarged, and lower intercalated population), but this does not shift the preference toward solely adopting the NMR structures.

The CHARMM36 nucleic acid force field shows significantly less conformational variability in the predicted GACC ensemble than is observed with any of the AMBER force field variants tested here. The main structure sampled, accounting for ∼45% of the population and having an RMSD of ∼3.4 Å from A-form RNA, is extended similar to the orientation of the NMR major structure. However, in this structure the G1 base is flipped around its χ torsion to populate *syn* values instead of the canonical *anti*, and the entire structure is overrotated. Though a low percentage of the canonical NMR major population is found, the NMR minor structure is exclusively dominated by incorrect *syn* distributions of χ, and no population of the canonical is seen. Additional clusters with low population include inverted and intercalated structures, accounting for <5% of the total ensemble.

The ff99 + Chen–Garcia modifications, intended to reduce the over-stacking problem seen in AMBER ff99 simulations, do indeed reduce the population of structures which could be characterized as stacked—the intercalated and inverted clusters, specifically, are populated far less than in the AMBER ff12 ± vdW_all/bb_ or ff99 + χ_Yil_ simulations. However, the main cluster sampled includes a base pair between residues G1 and C3, and can still be considered overly compact when compared to the NMR major structure. The ff99 + Chen–Garcia modifications sample low populations of the NMR major and minor structures, showing these as equivalently sampled with respect to each other (and also with respect to the intercalated structure in one run).

### All of the tested force fields predict a more limited conformational ensemble for the r(CCCC) tetranucleotide

**FIGURE 4.**
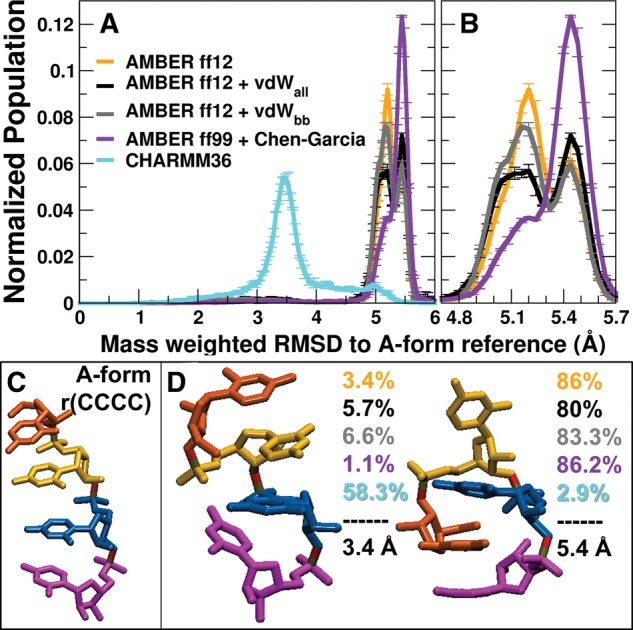
M-REMD RMSD to A-form Reference for r(CCCC). (*A*) M-REMD RMSD histogram profiles for r(CCCC) in five force fields. The averages of two runs per force field are shown, with error bars as standard deviation between runs. (*B*) Close-up of AMBER force fields, 4.8–5.7 Å. (*C*) A-form reference structure. (*D*) *Top* two clusters from combined cluster analysis and their populations in each force field, colored to match the force field designations from the *top* histogram plot. The mass-weighted RMSD to the A-form reference is provided for each structure.

In Figure 4A,B, the mass-weighted RMSD from a canonical A-form reference is shown for ensembles of CCCC from M-REMD simulations with five force fields. In each case, the top two clusters from combined cluster analysis, which account for a majority of each force field's population (Fig. 4D), differs significantly from the experimental structure (Fig. 4C). Of the force fields tested, the closest structure in RMSD space is found by CHARMM36, which deviates ∼3.4 Å from an A-form reference. Detailed cluster analysis results, shown in Supplemental Table 2 and Supplemental Figure 3, show that CHARMM36 finds the highest amount of the low populated A-form-like cluster. The top cluster accounts for 58% of the ensemble's population, and though it is an extended structure, it does not reproduce a majority of experimental NOEs (shown in Supplemental Table 3; Tubbs et al. 2013). Interestingly, this extended structure is very similar to the extended GACC structure determined by CHARMM36, shown in Supplemental Figure 4. As shown in Supplemental Figure 5, these similar structures occupy backbone dihedral values that are shifted from the NMR expected values. AMBER ff12 ± vdW mods populate an intercalated structure similar to the intercalated GACC structure, though the C2 base is extruded. This indicates that the vdW modifications did not help the CCCC ensemble as they did for the GACC ensemble. Additionally, the ff99 + Chen–Garcia modifications, intended to decrease stacking interaction favorability, similarly did not improve the CCCC ensemble. We hypothesize that this is due to the compactness of four pyrimidine bases versus the mixed purine/pyrimidine GACC system. Hydrogen bond analysis of the ff12 cluster trajectories for the intercalated structure reveals a loss of two hydrogen bonds in the GACC structure that are each 48% occupied in the CCCC structure, showing a subtle destabilizing effect though the representative structures themselves overlap with low RMSD (Supplemental Table 4; Supplemental Fig. 6).

The bimodal nature of the AMBER force field RMSD profiles, shown in Figure 4B, can be isolated with alternate cluster analysis, where the minimum distance between clusters is decreased from 0.9 Å to 0.5 Å. In doing so, we find two highly populated representative structures, shown in the detailed cluster analysis in Supplemental Figure 3. The most populated cluster's representative structure is completely intercalated, while the second most populated cluster's representative structure is intercalated with C2 extruded, as in the previous clustering results. The representative structures from these clusters have 5.2 Å and 5.5 Å RMSDS to the A-form structure, respectively. The intercalated structure accounts for ∼52%–53% of the ff12 and ff12 + vdW_bb_ ensembles, while the intercalated structure with the C2 residue unstacked is sampled ∼27%–30% of the ensemble. Adding increased vdW radii in ff12 + vdW_all_ system resulted in a more even split of ∼41%–33% intercalated/intercalated C2 extended. Interestingly, for AMBER ff99 + Chen–Garcia, this ratio is almost reversed—there is a slightly higher (∼61%) percentage of the population which occupies the intercalated C2 unstacked structure.

### Ensembles of the UUCG tetraloop are highly dependent on force field

Given the success of the M-REMD approach in generating well-converged conformational ensembles of the tetranucleotides, and access to the large-scale Blue Waters Petascale Resource, the larger UUCG tetraloop structure was investigated via M-REMD. In these simulations, the two terminal base pairs of the stem were restrained with Watson–Crick hydrogen bonding restraints. This was done in order to decrease the amount of total sampling that would be necessary to converge the unfolded stem structure, and focus our efforts on converging the loop ensemble. In each M-REMD simulation, 360 replicas were used and simulations were run out to >2 μsec per replica, aggregating 0.72 msec of sampling. Due to the large amount of sampling performed for each force field, and the fact that simulations were performed in duplicate, we were necessarily limited to a subset of the force fields tested in the tetranucleotide systems and focused our efforts on AMBER and AMBER-compatible modifications. Analyses of independent runs for each system were performed to gauge convergence and are shown in Supplemental Figure 7. The last 1 μsec of each simulation was used for analysis.

**FIGURE 5.**
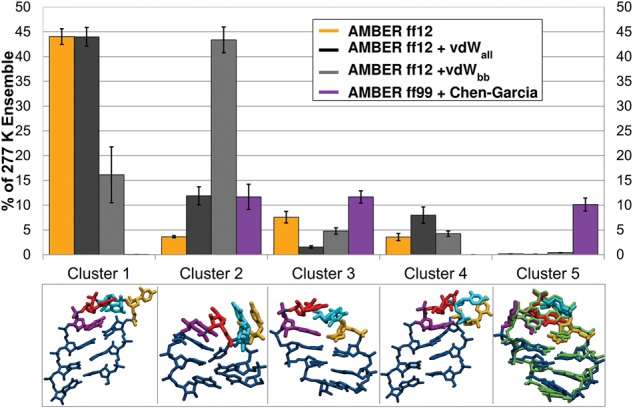
Combined clustering results for UUCG tetraloop simulations. Cluster number is shown on the *x*-axis and percentage of each simulation's 277 K ensemble is shown on the *y*-axis. Representative structures are shown *below* the bar graph. The native structure found in cluster 5 is shown overlapped with the NMR structure (in green).

Figure 5 shows the results of combined clustering, where we assign clusters based on the cumulative data set (here, the last 1 μsec per run, two independent runs per force field) to compare populations of representative structures found by each force field. The AMBER ff12 and ff12 + vdW_all_ force fields both sampled ladder-like structures at 277 K, seen in Clusters 1 and 4, to a significant extent—they are the majority of the ensemble population in each force field, and the remaining structures identified in combined clustering each account for <10% of the population. Cluster 1 is also highly sampled by ff12 + vdW_bb_, indicating that limiting the vdW modifications to the backbone does have an effect on the ensemble. Limiting the vdW modifications to the backbone in the ff12 + vdW_bb_ system shifted the majority of the structures sampled to Cluster 2. Cluster 2 is interesting in that its primary characteristic appears to be the stacking of the loop bases with each other and with the following stem. This stacked structure is likely stabilized by directly coordinating a K^+^ ion to the bases and backbone (Supplemental Fig. 8). The populations of Cluster 2 found in ff99 + Chen–Garcia and ff12 + vdW_all_ are roughly identical, while the highest population of Cluster 2 is found in ff12 + vdW_bb_. This is interesting given that the former force field has decreased vdW radii to penalize stacking interactions in particular, and the latter force fields have increased vdW radii, and indicates that modifying any vdW radius can alter interactions with ions and solvent. The ff99 + Chen–Garcia structures populate Clusters 2, 3, and 5 each ∼10%–15% of the ensemble, indicating that these structures are equally favorable. Clusters 3 and 5 adopt the most native-like characteristics. Cluster 3 deviates from the NMR structure in the χ dihedral of the G_L4_ base, which is flipped *anti* in this structure instead of *syn*, precluding the correct orientation of GU base pair formation. Additionally, the U_L2_ is rotated toward the opposite side of the loop from its position on the native structure. Cluster 5 is the native UUCG loop structure, which is most highly populated by ff99 + Chen–Garcia, but also accounts for a small percentage of the ff12 ensemble. The native structure is seen in <1% of the population in ff12 + vdW_all_ mods.

We further investigated the ladder-like structures populated by AMBER ff12, ff12 + vdW_all_, and ff12 + vdW_bb_ mods (Fig. 5, Clusters 1 and 4) at 277 K by measuring the RMSD from a representative structure with a ladder-like stem for each force field, as seen in Supplemental Figure 9. The RMSD shows a significant population of structures in the ensemble that adopts ladder-like stems. This is especially interesting since these three force fields contain χ fixes that attempt to explicitly address and overcome this issue. The ladder-like stem was identified in simulations of hairpin ribozymes using ff99 + parmbsc0 (Mlýnský et al. 2010). We believe this to be a completely nonphysiological conformation, and suggest that its population is purely a result of force field bias. Corrections based on high-level QM calculations and taking into account solvent effects were released in 2011 (Zgarbová et al. 2011), have been incorporated in all subsequent AMBER ffs (i.e., they are among recommended parameters for all RNA systems), and we have since verified that they were appropriately applied in these M-REMD simulations. This might suggest that the prevalence of ladder structures at 277 K could be a function of our biasing potential; decreasing the dihedral force constant promotes transitions to the ladder form, which becomes a trapped structure at 277 K. However, unrestrained long MD simulations up to 500 nsec in length at both 277 K and at 300 K show reluctance of the stem to transition back to a helix, at least on this timescale (Supplemental Fig. 10). We believe that the reappearance of the ladder-like structure is due to the increased amount of sampling being performed in the M-REMD simulations. Validation of the χ parameters was performed on the order of 50 μsec aggregate sampling from T-REMD simulations. In our M-REMD simulations we see the appearance of the ladder as the top cluster at 277 K after 500 nsec per replica, or the equivalent of 145–180 μsec aggregate sampling.

**FIGURE 6.**
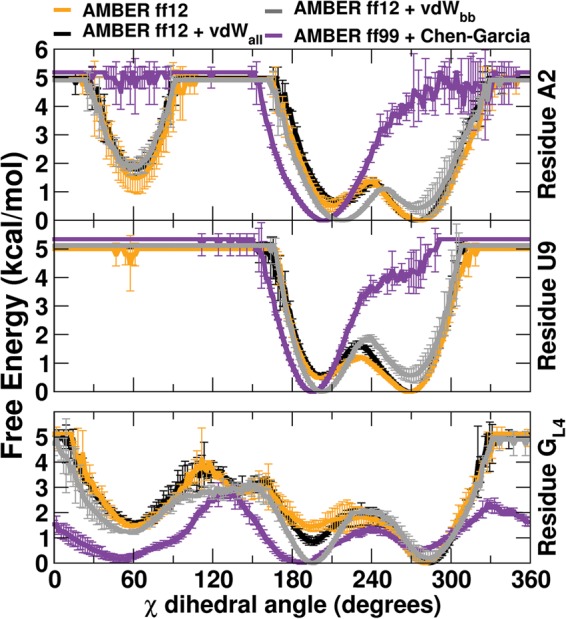
Free energy versus χ dihedral angle distribution at 277 K for the A:U stem base pair (*top*) and G_L4_ residue (*bottom*) for four force fields. *Syn* minimum is 60°, *anti* minimum is 180–240°, and high *anti* is 270°. Data are the average of two independent runs with error bars representing standard deviation.

Interestingly, ladder-like stems are not sampled at all in the UUCG ensemble with the ff99 + Chen–Garcia modifications. This indicates some deviation in χ parameters since a shift to the high-*anti* region (χ ≥ 270°) is a main indicator of the ladder-like structure. The χ parameters were refit in the Chen–Garcia modifications after modifying the van der Waals radii, and are not the standard ff99 χ parameters that were in use when the ladder-like structures were initially observed (Mlýnský et al. 2010). Figure 6 shows the free energy for χ rotation of stem base pair A2:U9 (top two graphs) and G_L4_ (bottom graph) for each of the force fields. The χ dihedral angle profiles at 277 K for ff12 ± vdW_all_ mods show slight preference for the noncanonical high-*anti* region, and also favor the canonical *anti* region. The ff12 + vdW_bb_ mods show a slight shift in the minima from high-*anti* to the canonical-*anti* region. The Chen–Garcia modifications, on the other hand, penalize the high-*anti* region. Additionally, the A2 residue shows a distinct minimum at the *syn* orientation for the AMBER ff12 variants though this should be penalized in a canonical helix, such as the UUCG stem. Though the canonical stem χ dihedral values were best maintained by ff99 + Chen–Garcia, the G_L4_ residue dihedral angle profiles show significantly lower barriers to rotation between *syn* and *anti* at 277 K (Fig. 6, bottom). This could contribute to the ff99 + Chen–Garcia ensemble populating loop structures where G_L4_ is *anti* rather than *syn*, seen in Figure 5, Clusters 2 and 3.

Though the 300 K ensembles are slightly less well-converged than the 277 K ensembles based on the correlation between independent runs’ cluster populations, representative structures are significantly different than the structures populated at 277 K. Shown in Supplemental Fig. 11, *none* of the 300 K ensemble representative structures have a ladder-like stem. The top cluster, seen in >20% of ff12 + vdW_all/bb_ and >10% of ff12, is similar to the native UUCG tetraloop, though the U_L2_ base has intercalated between the U_L1_:G_L4_ trans wobble base pair, breaking those hydrogen bonds. The G_L4_ base χ dihedral angle remains in the *syn* conformation. Clusters 2 and 5, populated by ff12 ± vdW_all/bb_ modifications, show solvent exposed loop bases participating in nonnative stacking interactions. Interestingly, ff99 + Chen–Garcia populates a structure in which hydrogen bonds between the loop residues and stem have formed.

## DISCUSSION

UUCG tetraloop simulations on nanosecond and microsecond timescales show instability in the loop region, and indicate significant rearrangements are possible on these shorter timescales. In simulations of short duration, meant to reproduce short-timescale dynamics and sampling near the native structure, current force fields have been shown to quantitatively reproduce experimental measurements (Giambaşu et al. 2015). These results are supported in this study, where the stable15 simulations maintained the native UUCG tetraloop contacts. However, once structures deviated from native contacts they did not return, as shown by the unstable5 simulations on the 100-nsec timescale as well as the simulations on the microsecond timescale. The inability of the force field to reform native contacts for this tetraloop, known experimentally to be extremely stable, was worrisome and led us to investigate these limitations further using enhanced sampling methods.

Tetranucleotide ensembles provide interesting insight into force field deficiencies, initially described in Yildirim et al. (2011) and Henriksen et al. (2013). These ensembles can be very conformationally diverse, and initial results using modified χ parameters (ff99 + χ_Yil_) shifted the populations of the RNA to sample mostly NMR major and minor conformations on the 100-nsec timescale (Yildirim et al. 2011). Later, enhanced sampling identified four main structures in AMBER ff12, though convergence in T-REMD simulations remained elusive (Henriksen et al. 2013). Here, we have generated highly converged ensembles using both force fields, showing the overabundance of compact intercalated structures remains in AMBER ff12, and becomes the most sampled structure in ff99 + χ_Yil_. Noticeably, in the ff99 + χ_Yil_ ensemble the ratio of NMR major to NMR minor structures is the most representative of experiment, yielding a 2.5:1 ratio compared to a 3:1 experimental ratio. Though larger problems with the force field remain, these χ modifications work effectively to shift the ensemble closer to reproducing experiment.

The CHARMM36 force field does return extended tetranucleotides as the representative structures in the GACC and CCCC ensembles, though these structures have significant deviations from the experimentally predicted structures and do not fit a majority of the experimental NOEs. χ populations favoring *syn* over *anti*, and high populations of noncanonical torsion values indicate potential problems with the dihedral angle force field terms. The overlap between representative GACC and CCCC structures points to a systematic error which exists independent of sequence.

While the ff12 + vdW_all/bb_ modifications shifted the GACC ensemble from over-stacked structures toward favoring the NMR major and minor as the top two clusters, this was not the case for the CCCC tetranucleotide ensemble. We hypothesize that this is due to the ability of CCCC to form a compact, highly favorable network of hydrogen bonds between nucleobases and the phosphate backbone in addition to the stacking interactions. Neither the ff12 + vdW_bb_ modifications, which increased the phosphate oxygen radii (but left base vdW radii intact), nor the ff99 + Chen–Garcia modifications to the nucleobase heavy atom vdW radii (but left the backbone untouched), are enough to destabilize this network in favor of extended structures. In GACC, the presence of two purine bases limits the degree to which the tetranucleotide can be compacted, resulting in a less stable hydrogen bond network which can be more easily disrupted.

The ff99 + Chen–Garcia modifications result in overly hydrogen bonded structures for GACC as well as CCCC. Interestingly, the top CCCC structure seen with this force field has the C2 unstacked but the core of the intercalated structure intact. This makes sense in the context of the intended ff99 + Chen–Garcia modifications—reducing stacking allows more flexibility at position C2 in particular, as it is not in a position to form many hydrogen bonds. The hydrogen bonds stabilizing the rest of the intercalating C's are not affected by the modifications from this force field, so the structures remain similar to those sampled by the other AMBER force fields.

In the UUCG restrained tetraloop system, sampling only loop conformations required over half a millisecond worth of aggregate sampling to converge the two independent simulations. Preliminary data on folding the tetraloop shows that stem formation alone, using these M-REMD simulation dimensions, is predicted to take well over 800 nsec per replica (using 576 replicas, Supplemental Fig. 12). The most recent results for UUCG tetraloop folding were drawn from T-REMD simulations averaging ∼500 nsec per replica, or ∼58-μsec aggregate sampling (Chen and García 2013; Kührová et al. 2013). It is therefore reasonable that corrections validated for shorter timescale simulations could prove problematic when the ensemble is more thoroughly sampled. It also serves to highlight the usefulness of generating the entire loop ensemble since we can more accurately pinpoint problems with current parameters.

Though the stem was restrained, the loop sampled similar conformational space as reported in Kührová et al. (2013). The increased amount of sampling performed here gives a better picture of the preference for UUCG to adopt certain structures in the individual force fields tested. Transitions to ladder-like structures were highly populated in both AMBER ff12 ± vdW_all_ modifications at 277 K, as shown by the free energy dihedral angle profiles for the central stem A:U base pair. AMBER ff12 + vdW_bb_ modifications slightly, but reproducibly, penalize the high-*anti* region and shift the minima back to the canonical *anti* χ dihedral, reducing the population of structures adopting a ladder-like stem. These same profiles showed that the ff99 + Chen–Garcia χ modifications more heavily penalized the high-*anti* region of the distribution, and ladder structures were not found using this force field. However, these χ modifications also lowered the free energy barrier to rotation around the χ dihedral angle for the G_L4_ residue, adversely affecting the residue's preference for adopting the *syn* conformation. Of the representative structures shown in Figure 5, ff99 + Chen–Garcia identifies three as almost identically populated, two of which have G_L4_ anti χ dihedral angle values, and one that is the native structure. The most populated structures for AMBER ff12 ± vdW_all_ modifications maintained the correct orientation of the U_L2_ and C_L3_ bases, though G_L4_ was flipped anti and U_L1_ was extruded.

**FIGURE 7.**
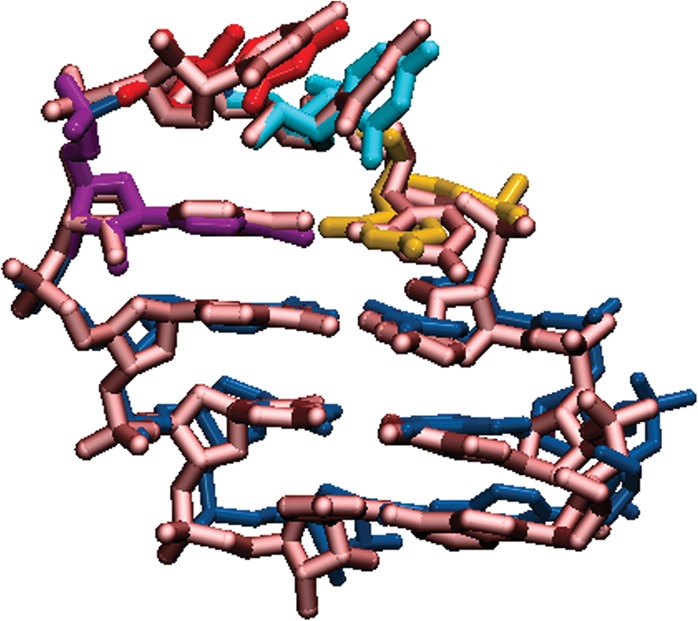
Overlap of Anton Run 2 final structure (metallic red) with representative structure from Cluster 3 of combined cluster analysis at 277 K (blue stem and colored UUCG loop bases). All atom RMSD is 0.7 Å.

When comparing UUCG's M-REMD ensemble to the initial long MD simulations, key structural features that appear in the long MD simulations become major characteristics of the ensemble. In most of the unstable5 simulations, the G_L4_ has broken the *trans* wobble base pair and is extruded into a solvent exposed position. We propose that this is an intermediate in rotating around the G_L4_ χ dihedral from *syn* to *anti*, and a precursor to reforming the G–U base pair after G_L4_ has flipped to an *anti* orientation, as seen in Cluster 3's representative structure from the 277 K ensemble. Other representative structures occupy G_L4_ *anti* χ dihedral angle values as well (Clusters 1, 3, and 4 are high *anti* while Cluster 2 is *anti*). The final structure of one of the unstable5 simulations matched a low populated representative cluster's structure found during clustering of the ff12 M-REMD simulations. Interestingly, when the MD simulations were extended to longer than 5 μsec using Anton, the second run became trapped in a structure matching Cluster 3 from the 277 K ensemble's combined clustering results, shown in Figure 7. These results illustrate how force field inaccuracies can manifest in short (nsec–μsec) timescale simulations, which can severely limit their predictive abilities. A caution with all of these force fields is that as sampling is increased, the probability of deviating from the expected RNA structures will also increase.

Complete convergence of any simulation is, of course, unprovable. Common assessments of convergence instead focus on reporting the minimized differences between at least two independent simulations. Simulations can start from different initial velocities or solvent/ion distributions and be considered independent, or, as reported in this study and others, from completely different starting structures, commonly from folded and unfolded or different ensembles of the folded structure (Chen and García 2013; Kührová et al. 2013; Bergonzo et al. 2014). Additional measures of convergence examined in this study include the use of different methods of biasing the M-REMD simulations, resulting in equivalent sampling between two runs. Differences between ensembles generated by each force field are larger than any differences between two independent runs, leading us to believe these simulations are sampling nearly identical conformational space. In our experience, once several measurements (e.g., overlap of RMSD/PC projection histogram distributions, cluster populations, etc.) from independent M-REMD simulations all agree, all major conformations appear to be sampled (Bergonzo et al. 2014; Roe et al. 2014). That is, while we cannot rule out conformations that may be accessible on second or longer timescales, it is unlikely that a highly populated conformation was not sampled during these M-REMD simulations which spanned high temperature and reduced barriers to conformational transitions.

## CONCLUSIONS

The highly sampled ensembles of small RNA motifs presented here summarize problems with the current nucleic acid force fields. No single force field tested has demonstrated a robust performance for these RNA systems. The deficiencies present in each potential function range from shifts in the backbone dihedral angles to nonnative values (CHARMM36), to an imbalance between modified and unmodified vdW (ff12 + vdW, ff99 + Chen–Garcia), to over-populated intramolecular hydrogen bonding, to problems with the χ dihedral angle distribution (all force fields sampled). Current study is underway in many groups to address these problems. Since these force fields are all widely used, it is important to understand their performance so that errors in the potential function are not deemed relevant in larger systems or in a biological context.

## MATERIALS AND METHODS

The UUCG tetraloop structures for long MD simulations were built using the tleap program in AMBER12 (Case et al. 2012). Each of the 20 NMR models deposited in the PDB (ID:2KOC) was used as initial starting structures for simulation, with the sequence r(GGCAC-UUCG-GUGCC) (Nozinovic et al. 2010). The 5′ phosphate group was removed in order to fit the standard AMBER library definition. The RNA was parameterized using AMBER ff12, which combines ff99 (Wang et al. 2000) with Barcelona modifications to the α/γ torsions (Pérez et al. 2007b) and updated χ parameters (Zgarbová et al. 2011) (refer to Table 1 for force field details). Each model was solvated in an ∼20 mM KCl solution by adding 4983 TIP3P water molecules, 15 K^+^ cations, and 2 Cl^−^ anions in the shape of a truncated octahedron (Jorgensen et al. 1983). The final positions of the ions were randomized with water molecules to prevent any initial bias. Ion parameters were taken from Joung and Cheatham (2008).

Prior to production simulation, each system was minimized and equilibrated. Minimization was performed with 25 kcal/mol^−1^ Å^−2^ positional restraints on the RNA and consisted of 1000 steps using the steepest decent algorithm followed by 1000 steps using the conjugate gradient algorithm. The system was initially heated from 10 to 150 K over the course of 100 psec in the canonical ensemble (NVT) using the Langevin thermostat with a collision frequency of 2.0 psec^–1^ and an integration time step of 1 fsec (Loncharich et al. 1992). RNA positional restraints were 25 kcal/mol^−1^ Å^−2^. Further heating from 150 to 298 K was accomplished over 100 psec in the isothermal–isobaric (NPT) ensemble at 1 atm using the same thermostat and time step as the previous step. Constant pressure was maintained with the weak-coupling algorithm (Berendsen et al. 1984). RNA positional restraints were 5 kcal mol^−1^ Å^−2^. Equilibration at 1 atm and 298 K was performed for 5 nsec in the NPT ensemble using a 2-fsec time step and the same thermostat and barostat settings as before. RNA positional restraints were 0.5 kcal mol^−1^ Å^−2^. Production simulations were performed at 1 atm and 298 K using the weak-coupling algorithm for both the barostat and thermostat and a 2-fsec time step.

Production simulations were 200 nsec for each of the 20 models using M2090 nVIDIA GPUs available on the Keeneland system (Towns et al. 2014). Five additional and longer simulations were performed under similar conditions. Three were based on the first three model structures using the same force field, however with 4416 TIP3P waters, 32 Na^+^ and 19 Cl^−^ ions in random initial positions. A slightly different equilibration procedure, equivalent to our previous study, was performed (Shao et al. 2007) with production simulations on both GPU and CPU resources. The remaining two longer MD simulations were performed on the special purpose supercomputer for molecular dynamics at PSC, Anton, built by DE Shaw Research, Inc. (Shaw et al. 2008). A different stem was used with the sequence r(GGC-UUCG-GCC); this shorter stem substitutes a GC base pair for the native AU base pair to theoretically improve stability of the stem and therefore the folded loop structure with fewer atoms. This particular stem was chosen as part of a systematic study of all tetraloop structures found in the PDB where the loop structure was grafted onto a short, but stable, G-rich stem structure for systematic validation and assessment of the ability of various force fields available at the time to model tetraloop structure. These studies, initiated in the mid 2000s, did not well reproduce tetraloop structure, lacked complete sampling, and were never published. When access to Anton became available, the model UUCG tetraloop on the r(GGC) stem was simulated on Anton. The structures were solvated with 4000 TIP3P waters in a cubic box (as required of Anton at that time) with 24 Na^+^ and 15 Cl^−^ ions placed randomly. The equilibration protocol of Shao et al. (2007) was performed on CPUs. Production MD simulations were run using multiple different versions of the Anton software and microcode (initially 2.4.1 and then 2.4.5). Further details for running on Anton using Desmond are provided in the Supplemental Information.

M-REMD simulations were performed using two dimensions, temperature and (unless otherwise noted) dihedral force constant scaling (referred to as DFC). For the M-REMD simulations of UUCG, the first structure of the UUCG tetraloop was taken from PDBID 2KOC and truncated to 10 bases, containing a 3-bp stem and the four membered loop (Nozinovic et al. 2010). The sequence used in these simulations was r(CAC-UUCG*-*GUG), and bases were renumbered 1–10, where bases 4–7 indicate the UUCG loop and may also be denoted by the common nomenclature U_L1_,U_L2_,C_L3_,G_L4_. Since we wanted a faster way to converge the loop ensemble, and also to avoid the long timescales required to sample the unfolded states, Watson–Crick restraints from the available NOE data were enforced for the two terminal base pairs of the stem, leaving the tetraloop and its capping C–G base pair free to move. These restraints were enforced during the initial minimization and equilibration, using a force constant of 20 kcal mol^−1^ Å^−2^. Minimization and equilibration were performed as previously described, in the same manner as the tetranucleotide systems (Henriksen et al. 2013).

To determine the proper spacing of Hamiltonian replicas for the UUCG M-REMD simulations, first several short MD simulations were performed scaling the DFC in 0.1 intervals from 0.9× to 0.1×. The resulting scale factor versus average dihedral energy was plotted and fit with an exponential curve. The equation for this fit was then used to determine an exponential interval overlap in dihedral energy space (as opposed to a linear overlap). The resulting scale factors were 1.0, 0.9, 0.81, 0.73, 0.66, 0.59, 0.53, 0.48, 0.43, 0.39, 0.35, 0.31, and resulted in a uniform acceptance rate of 50%. The temperature range was calculated using an online generator for an acceptance rate of 30%, and consisted of 30 temperatures spanning from 277 to 400 K (Patriksson and van der Spoel 2008). This yielded 360 replicas for the UUCG M-REMD system.

The GACC tetranucleotide was built, minimized, and equilibrated as previously described using each of the force fields that appear in Table 1 (Henriksen et al. 2013; Bergonzo et al. 2014). Structures built natively in CHARMM36 were converted to AMBER topology files using CHAMBER (Crowley et al. 2009). Structures built using AMBER ff99 applied the Chen–Garcia modified vdW radii for nucleobase atoms using a frcmod file in teLeap. Modifications to the off-diagonal nucleobase-water vdWs for this same force field were performed using *parmed.py*. These files are provided in the Supplemental Information. A frcmod file was used to modify the van der Waals parameters for O2, OS, and OH atom types for structures built with AMBER ff12 + vdW_all_, and the original van der Waals radius and ε values were restored for the O4′ atom (which is an OS atom type) only in the AMBER ff12 + vdW_bb_ systems using *parmed.py* (Steinbrecher et al. 2012). These files are provided in the Supplemental Information. CCCC tetranucleotide structures were built using the sequence command in tleap, following the same methods described previously (namely, neutralizing charge with Na^+^ during the addition of 2500 solvent molecules) (Henriksen et al. 2013; Bergonzo et al. 2014).

For each of the M-REMD simulations of CCCC and all GACC systems, 24 temperature replicas spanning 277–396 K and eight Hamiltonian replicas scaling the DFC (except ff99 + χ_Yil_, discussed below) from 1.0× to 0.3× in intervals of 0.1 were used resulting in a total of 192 replicas, matching previous M-REMD simulations (Bergonzo et al. 2014). For the M-REMD simulations of GACC with ff99 + χ_Yil_, the same temperature range was used along with eight Hamiltonian replicas using varying degrees of accelerated MD dihedral boost (see Supplemental Table 5 for boost parameters used) for a total of 192 replicas. Previous work has shown that dihedral force constant scaling and accelerated MD M-REMD simulations of GACC converge in a similar amount of simulation time (Roe et al. 2014).

M-REMD simulations were carried out with the *pmemd.cuda.MPI* module of the AMBER14 suite of programs (Case et al. 2014). Production dynamics for each replica were carried out in the NVT ensemble. The Langevin thermostat was used to regulate temperature with a collision frequency of 2 psec^−1^ (Loncharich et al. 1992). The “ig = -1” option was set to automatically generate random seeds for each restart to avoid synchronization effects (Sindhikara et al. 2009). An integration time step of 2 fsec was used. Exchanges were attempted every 1 psec. The nonbonded direct space cutoff was set to 8.0 Å, and default AMBER 14 particle mesh Ewald settings were used for reciprocal space calculations. SHAKE was used to constrain bonds to hydrogen (Ryckaert et al. 1977). Snapshots from the MD simulations were written every 10 psec to the trajectory file. Simulations were performed on the NCSA Blue Waters Petascale Resource. Production M-REMD simulations were 300 nsec per replica for tetranucleotide systems, and at least 2 μsec per replica for the UUCG tetraloop system. For all tetranucleotide systems and for ff12 + vdW_all_ mods and ff12 in the UUCG tetraloop system, the first M-REMD runs were initiated with all replicas at the equilibrated structure. To seed the second set of M-REMD runs for these systems, the initial structures were simulated at their respective Hamiltonians and temperatures, starting from new initial velocities, for 5 nsec without exchanging. For the UUCG tetraloop simulations with ff99 + Chen–Garcia, the first M-REMD simulation started from the ff12 + vdW mods run 2 1.8-μsec ensemble. The second M-REMD simulation started from the ff12 run 1 1.45-μsec ensemble.

For the UUCG M-REMD simulations, hydrogen mass repartitioning was used to generate both AMBER ff12 ensembles and for one of the ff12 + vdW_all_ ensembles (Feenstra et al. 1999; Hopkins et al. 2015). In these simulations, *parmed.py* was used to repartition the hydrogen masses to 3.07 a.u., decreasing the mass on atoms to which the H is bonded by a respective amount. Mass repartitioning was not performed on TIP3P solvent molecules. This allowed us to use a 4-fsec integration time step in this system. All other simulation specifics remained the same.

All sorting of M-REMD trajectories and subsequent analysis was performed with a development version of CPPTRAJ (Roe and Cheatham 2013), now part of AmberTools14. Free energy profiles were calculated via histogram by using populations to calculate Gibbs free energy. Several scripts detailing how cluster analysis was performed are available in the Supplemental Information.

## SUPPLEMENTAL MATERIAL

Supplemental material is available for this article.

## Supplementary Material

Supplemental Material
