# Supplementary material for: Highly sampled tetranucleotide and tetraloop motifs enable evaluation of common RNA force fields
Source: RNA. 2015 Sep;21(9):1578–90. doi: 10.1261/rna.051102.115 (PMC4536319; doi:10.1261/rna.051102.115)
Supplement: Supplemental Material [file supp_21_9_1578__index.html]

Highly sampled tetranucleotide and tetraloop motifs enable evaluation of common RNA force fields — Highly sampled tetranucleotide and tetraloop motifs enable evaluation of common RNA force fields — Supplemental Material 

# Highly sampled tetranucleotide and tetraloop motifs enable evaluation of common RNA force fields

## Supplemental Material

**Files in this Data Supplement:**

- Supp Material.pdf
