# Supplementary material for: Highly sampled tetranucleotide and tetraloop motifs enable evaluation of common RNA force fields
Source: RNA. 2015 Sep;21(9):1578–90. doi: 10.1261/rna.051102.115 (PMC4536319; doi:10.1261/rna.051102.115)
Supplement: Supplemental Material [file supp_051102.115_SuppMaterial.pdf]

Supporting Information: Highly Sampled Tetranucleotide and Tetraloop Motifs Enable Evaluation of Common RNA Force Fields

Christina Bergonzo[†], *Niel M. Henriksen*[†,‡], *Daniel R. Roe*[†], *Thomas E. Cheatham III*[†]*

† Department of Medicinal Chemistry, College of Pharmacy, University of Utah, Salt Lake City, Utah 84112, United States

‡ Current Address: Skaggs School of Pharmacy and Pharmaceutical Sciences, University of California San Diego, 9500 Gilman Drive, La Jolla, California 92093-0736, United States

Contents

Supporting Figure 1: Loop region overlay of the final trajectory frames from the Stable-15 and Unstable-5 subsets on the NMR structure. 3

Supporting Table 1: Detailed cluster analysis for GACC. 4

Supporting Figure 2: Representative GACC structures. 5

Supporting Table 2: Detailed cluster analysis for CCCC.......... 6

Supporting Figure 3: Representative CCCC structures.......... 7

Supporting Table 3: Detailed NOE analysis for CCCC C36 top cluster trajectory. 8

Supporting Figure 4: Overlap of representative structure for most populated clusters in Charmm36 277 K ensembles of GACC and CCCC tetranucleotides.......... 9

Supporting Figure 5: C36 backbone dihedral angle distributions for all structures in the top cluster for GACC and CCCC tetranucleotide.......... 10

Supporting Table 4: Hbond analysis for ff12 intercalated cluster trajectories.......... 11

Supporting Figure 6: Overlap of intercalated representative structures from GACC and CCCC. 11

Supporting Figure 7a: Cluster correlation analysis for UUCG M-REMD simulations.......... 12

Supporting Figure 7b: Cluster correlation analysis for UUCG M-REMD simulations.......... 13

Supporting Figure 8: Close up of Cluster 2 from combined cluster analysis at 277 K coordinating a K+ ion. 14

Supporting Figure 9: RMSD to ladder reference. 15

Supporting Figure 10: Unrestrained MD simulations starting from ladder representative structure do not transition back to a helix in 500 ns. 16

Supporting Figure 11: Combined clustering results for UUCG tetraloop simulations at 300 K. 17

Supporting Figure 12: Cluster population vs. Time and cluster population correlation of unrestrained M-REMD UUCG folding simulations. 18

Supporting Text: Anton Simulation Details 19

Supporting Table 5: aMD parameters used in M-REMD simulations of GACC with ff99 + χ_{Yil}. 20

Supporting CPPTRAJ Script 1: Clustering command for analysis of GACC tetranucleotide simulations. 21

Supporting CPPTRAJ Script 2: Clustering command for analysis of CCCC tetranucleotide simulations. 21

Supporting CPPTRAJ Script 3. UUCG Combined Cluster Analysis 21

Supporting File: Frcmod.ff99CG – force modification file for building ff99 + Chen-Garcia LJ and dihedral modifications. 22

Supporting File: Parmed.py script for modifying off-diagonal LJ terms for ff99 + Chen-Garcia. 22

Supporting File: Frcmod.vdW – force modification file for building ff12 + vdW_{all} modifications. 23

Supporting File: Parmed.py script for modifying O4' atom from ff12 + vdW_{all} to default ff12, generating ff12 + vdW_{bb} modifications. 23

Supporting Figure 1: Loop region overlay of the final trajectory frames from the Stable-15 and Unstable-5 subsets on the NMR structure.

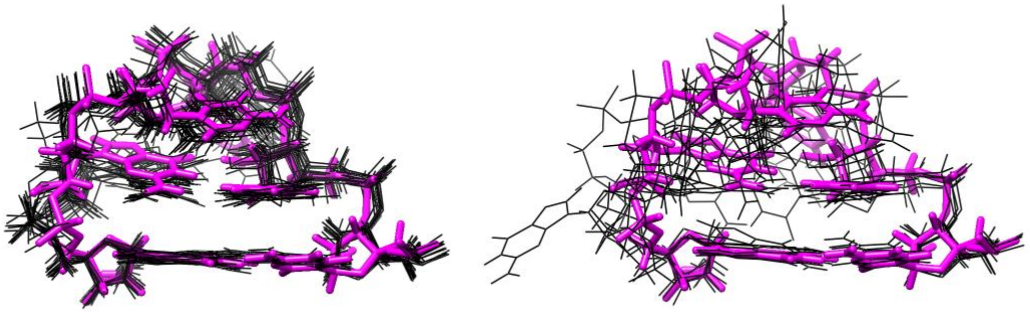

Loop region overlay of the final trajectory frames from the Stable-15 (left) and Unstable-5 (right) subsets on the NMR structure (model 1 PDB: 2KOC). Loop region consists of residues 5-10. The simulation structures are colored black. The NMR structure is magenta. Overlay was performed by RMS fitting to the NMR structure using residues 5 and 10

Supporting Table 1: Detailed cluster analysis for GACC.

277 K Replicas	ff12 run 1	ff12 run 2	ff12+ vdWall run 1	ff12+ vdWall run 2	ff12+ vdWbb run1	ff12+ vdWbb run2	C36 run 1	C36 run 2	ff99+ CG run 1	ff99+ CG run 2	ff99+Xyil run 1	ff99+ Xyil run 2
**Experimental Structures**												
A-form-major	15	16.6	26.7	27.9	32.5	30.6	9.1	7.8	7.9	8.4	19.1	19.2
A-form-minor	17.9	19.7	18.9	16.4	17.8	17.6	0	0	9	10.2	6.4	8.3
**Alternate NMR Major & Minor Conformations**												
A-form-major-1-syn	0	0	0	0	0	0	44.3	47.4	0	0	0	0
General A-form major	0.7	0.5	0.7	0.6	0.4	0.4	2.5	0.4	0.4	0	1	0.8
A-form-minor-1-syn	0	0	0	0	0	0	3.6	3.3	0	0	0	0
A-form-minor-4-syn	0	0	0	0	0	0	0	0	0	0	0.4	0.3
General A-form minor	0	0	0	0	0	0	1.8	5	0	0	0	0
**Conformations Classified as Intercalated**												
Intercalated-anti	14.1	14.5	10.5	9.4	14.4	15.3	0	0	6.7	5.7	22.2	21.7
Intercalated-2-anti-extruded	0	0.6	0	0	0	0	0	0	0	0	0.4	0
Intercalated-syn	3.2	3.5	2	0	3.3	3.1	0	1.1	1.8	1.5	17.8	12.1
Intercalated-syn-3-extruded	0.6	0.9	0	0.7	0.4	0	0	0	0	0	0.9	0.9
Intercalated-syn-2-extruded	1.4	1.2	1	0.8	0.6	0.8	0.5	0	1	0.8	0	0.7
Pre-intercalated	0.5	0	0	0	0	0	0	0	0.7	0	0.4	0
General Intercalated	0	0	0	0	0	0	3.6	1.8	0	0	0.3	0
**Conformations Classified as Inverted**												
Inverted-anti	0.7	0.7	1.7	2.3	1.7	1.6	0	0	0.6	0.5	0.7	0.7
Inverted-anti_2	0	0.5	0	0	0	0.4	0	0	0.4	0	0	0
Inverted-syn	7.4	5.5	3.7	4.9	5.1	4.7	1	1.1	2.1	1.9	12.3	11
Inverted-syn-conf2	0	0	0	0	0	0	2.4	2.3	0	0	0	0
**Conformations Classified as Base Paired**												
1_3-basepair	6	2.5	2.7	6.6	1.7	3.4	0	0.4	34.2	15.5	3.3	9.3
1_4-basepair	0.5	0.6	0	0.7	0.5	0	0	0	0	19.3	0	0
1_4-basepair-conf2	0	0	0	0	0	0	0.7	0	0	0	0	0
General Basepaired	0.4	0.3	0	0	0.5	0.8	0	0	0	0	0	0
**Conformations Classified as "Other"**												
1_3-stack	1.2	1.7	0.7	0	0	0.5	0	0	1.6	1.5	0	0
1_3-2_4-stack	1	1	1	0.6	0	1	0	0	0	0	0	0
1_4-stack (1_4-2_3)	0.4	1.5	0.5	0.6	0.4	0.3	0	0	0	0	0.7	0.5
3-extruded	1.1	1.4	1	1	1.4	1	0	0	1.4	1.3	1.4	1.2
2-extruded	1.3	1.5	0.6	0.5	0	0	0	0	0	0	0	0
1-4_hbond	0	0	0.9	0	0	0	0	0	0	0	0	0
Backbone hbonds	0	0	0	0	0	0	0	0	0	0	0.2	0
General hbonds	0	0	0	0	0	0	0.4	0.9	1.2	1.8	0	0

Supporting Figure 2: Representative GACC structures.

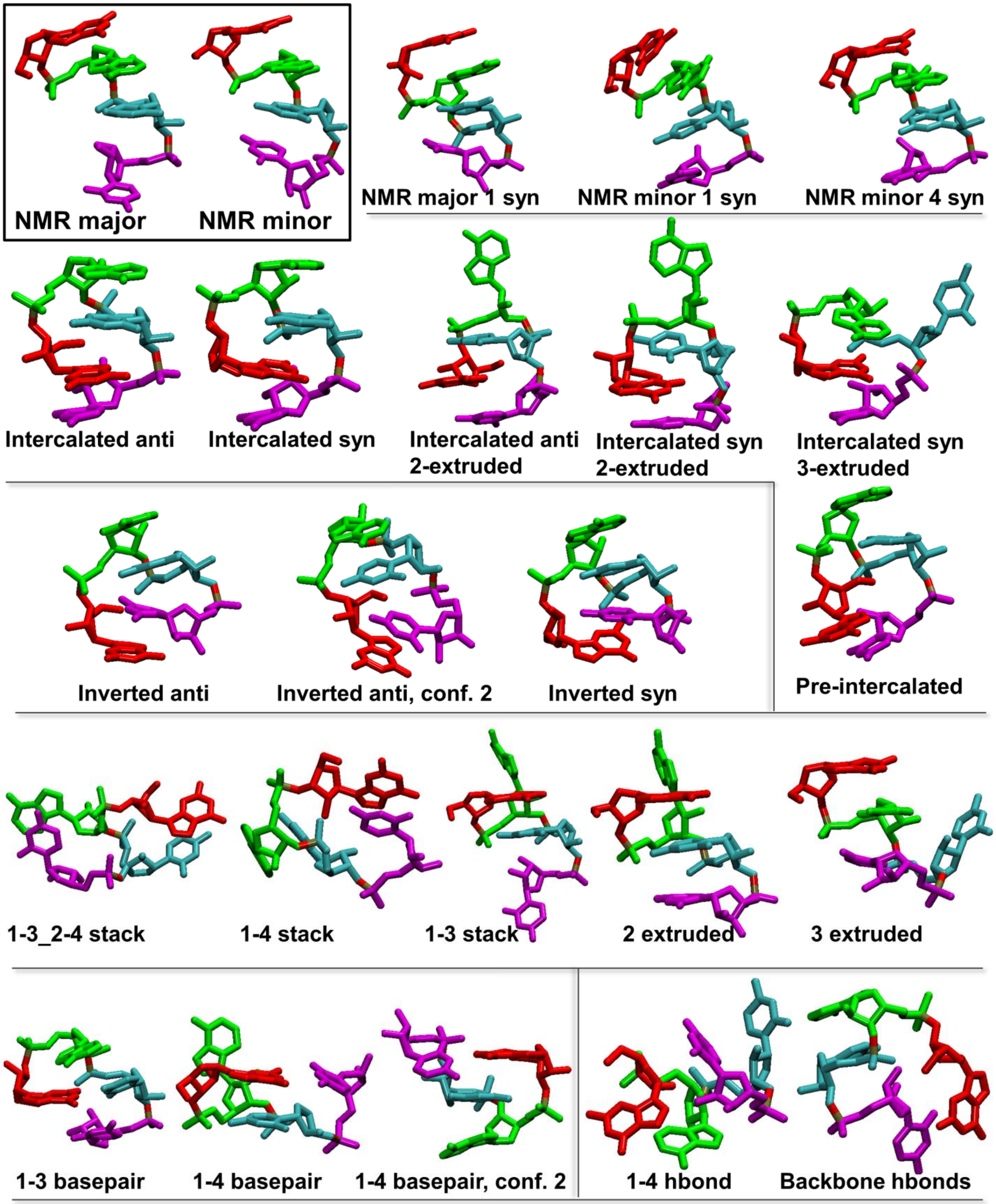

Supporting Table 2: Detailed cluster analysis for CCCC.

Representative Structure	Cluster	RMSD to A-form (Å)	ff12 run 1	ff12 run 2	ff12+ vdWall run 1	ff12+ vdWall run 2	ff12+ vdWbb run1	ff12+ vdWbb run2	ff99+ CG run 1	ff99+ CG run 2	C36 run 1	C36 run 2
Intercalated 2-extruded	0	5.3879	86.0%	86.0%	79.3%	80.7%	82.7%	83.8%	85.3%	87.0%	2.8%	3.0%
A-form hbonded	1	3.4186	3.4%	3.4%	5.5%	5.8%	6.7%	6.5%	1.1%	1.1%	58.4%	58.2%
A-form rotated	2	3.3183	3.4%	3.1%	3.7%	3.5%	3.8%	3.9%	1.2%	1.0%	3.6%	3.4%
Inverted	3	4.8372	0.2%	0.2%	0.6%	0.7%	0.6%	0.5%	0.3%	0.2%	3.6%	3.2%
2-3-4_stack	4	4.3178	0.0%	0.0%	0.0%	0.0%	0.0%	0.0%	0.0%	0.0%	1.9%	2.4%
1-3_stack	5	4.8614	0.4%	0.3%	0.6%	0.3%	0.2%	0.2%	0.9%	0.8%	0.0%	0.0%
1-3_2-4_stack	6	5.7911	0.2%	0.3%	1.2%	0.3%	0.3%	0.2%	0.2%	0.1%	0.0%	0.0%
1-3_2-4_stack, 1 syn	7	5.9179	0.1%	0.2%	0.7%	0.6%	0.2%	0.1%	0.5%	0.5%	0.0%	0.0%
**Above: dbscan minpoints 25 epsilon 0.9 rms :1@O2,H5,C1',P,:2@O2,H5,C1',P,:3@O2,H5,C1',P,:4@O2,H5,C1',P**												
Intercalated	0	5.1869	52.1%	53.7%	39.8%	43.1%	52.6%	52.1%	20.3%	20.5%	1.3%	1.5%
Intercalated 2-extruded	1	5.4948	30.4%	28.8%	34.2%	32.3%	26.7%	28.1%	59.8%	61.1%	0.0%	0.0%
A-form hbonded	2	3.4186	0.4%	0.6%	1.0%	0.8%	0.8%	0.8%	0.2%	0.3%	44.7%	43.6%
Intercalated 4-dangle	3	5.1468	1.1%	1.1%	1.7%	1.7%	1.4%	1.5%	0.7%	0.8%	0.3%	0.2%
A-form hbonded - rotate	4	2.3064	0.7%	0.7%	1.0%	1.4%	1.5%	1.7%	0.1%	0.1%	0.1%	0.1%
Pre-inverted/2-3-4_stack	5	4.859	0.0%	0.0%	0.0%	0.0%	0.0%	0.0%	0.0%	0.0%	1.7%	1.6%
**Above: dbscan minpoints 25 epsilon 0.5 rms :1@O2,H5,C1',P,:2@O2,H5,C1',P,:3@O2,H5,C1',P,:4@O2,H5,C1',P**												
Intercalated 2-extruded	0	5.3879	87.8%	87.6%	81.5%	83.1%	83.9%	85.0%	88.3%	90.1%	5.8%	5.9%
A-form hbonded	1	3.3446	4.6%	4.8%	7.2%	7.0%	7.9%	7.9%	1.5%	1.6%	62.5%	62.1%
A-form 4 dangle	2	3.5033	2.8%	2.4%	3.1%	3.2%	2.7%	2.8%	2.7%	2.2%	9.9%	10.1%
A-form/NMR	3	1.271	2.3%	2.3%	2.7%	2.6%	2.3%	2.0%	1.6%	1.3%	8.7%	8.2%
Pre-inverted/2-3-4_stack	4	4.4458	0.7%	0.9%	1.4%	1.4%	1.1%	0.9%	1.1%	1.1%	11.4%	11.4%
1-2-4_stack_3-extruded	5	4.0538	0.6%	0.9%	1.1%	1.0%	0.8%	0.7%	1.6%	1.0%	1.0%	1.1%
H-bonded	6	5.6714	0.7%	0.4%	0.9%	0.6%	0.6%	0.3%	2.0%	1.7%	0.7%	1.2%
1-3_2-4_stack, 1 syn	7	5.8688	0.6%	0.7%	2.3%	1.2%	0.7%	0.4%	1.3%	1.1%	0.2%	0.1%
**Above: kmeans clusters 8 rms :1@O2,H5,C1',P,:2@O2,H5,C1',P,:3@O2,H5,C1',P,:4@O2,H5,C1',P**												

Supporting Figure 3: Representative CCCC structures.

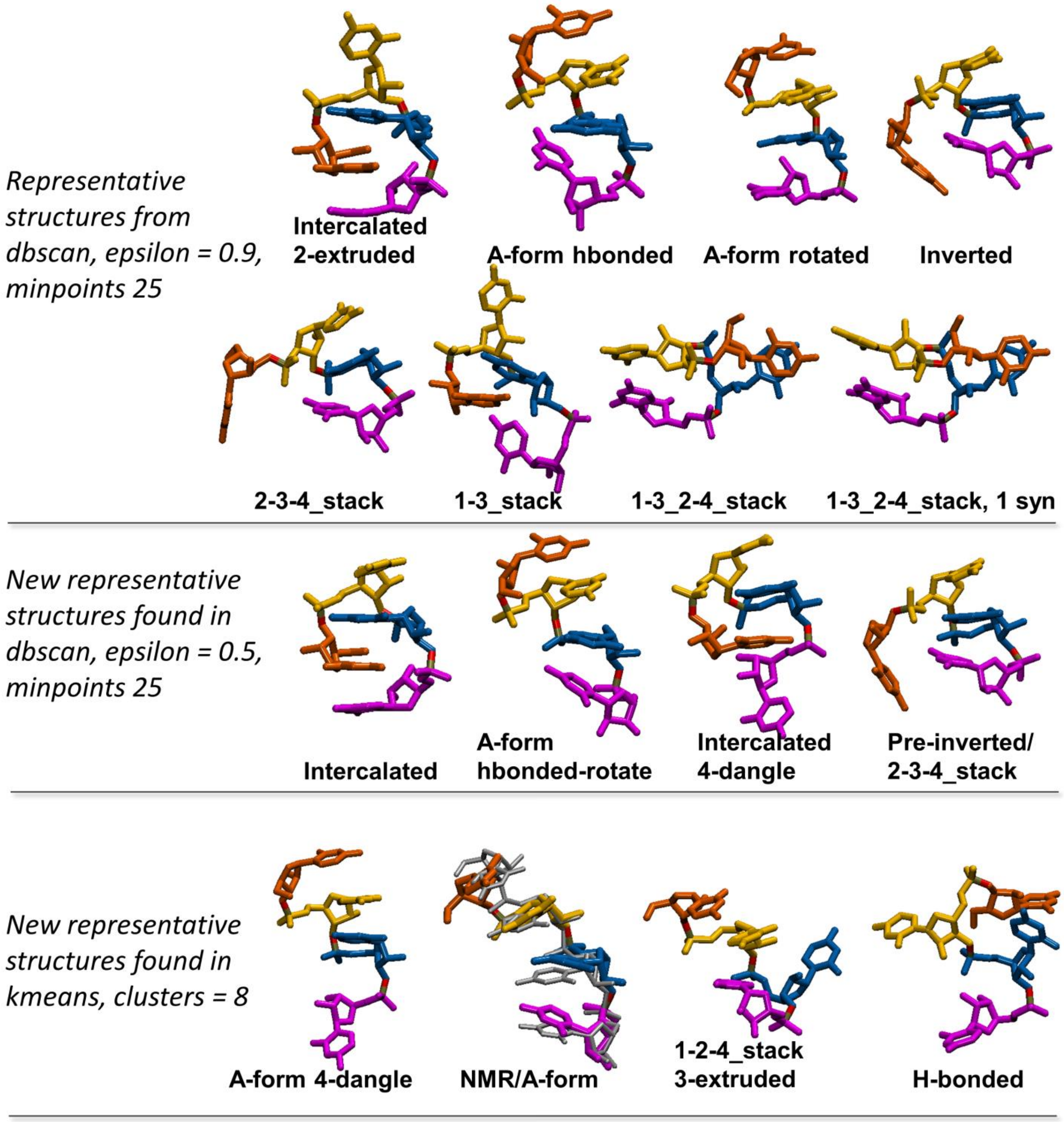

Supporting Table 3: Detailed NOE analysis for CCCC C36 top cluster trajectory.

Analysis performed using Cpptraj to calculate distances with r6 averaging. Average values which violate NMR bounds are shaded red. All values are in units of Å.

NOE	NMR value	NMR lower	NMR upper	C36 Run 1	C36 Run 2	C36 Avg	C36 StDev
:1@H2'-:2@H1'	4.21	3.77	4.61	4.1	4.1	4.1	0
:1@H2'-:2@H5'	2.82	2.61	2.98	2.51	2.51	2.51	0
:1@H5-:2@H5	3.79	3.45	4.07	3.61	3.61	3.61	0
:1@H6-:1@H1'	3.46	3.17	3.68	3.49	3.49	3.49	0
:1@H6-:1@H2'	3.38	3.1	3.6	3.82	3.82	3.82	0
:1@H6-:2@H5	3.66	3.34	3.91	3.41	3.42	3.415	0.005
:2@H5-:1@H2'	4.43	3.94	4.94	5	5.1	5.05	0.05
:2@H5-:3@H5	3.8	3.46	4.08	3.69	3.7	3.695	0.005
:2@H6-:1@H2'	2.73	2.52	2.88	3.07	3.12	3.095	0.025
:2@H6-:2@H1'	3.43	3.14	3.65	3.51	3.51	3.51	0
:2@H6-:2@H5''	3.49	3.2	3.72	4.12	4.12	4.12	0
:3@H3'-:4@H2'	3.32	3.05	3.52	5.36	5.29	5.325	0.035
:3@H5-:4@H5	3.67	3.35	3.93	3.72	3.72	3.72	0
:3@H6-:3@H2'	3.02	2.78	3.19	3.73	3.73	3.73	0
:3@H6-:3@H3'	2.62	2.42	2.77	2.53	2.53	2.53	0
:3@H6-:3@H5''	3.85	3.5	4.15	4.04	4.04	4.04	0
:3@H6-:4@H2'	4	3.62	4.34	6.49	6.47	6.48	0.01
:3@H6-:4@H5	3.84	3.49	4.13	3.22	3.21	3.215	0.005
:4@H1'-:3@H2'	4.39	3.91	4.88	4.37	4.4	4.385	0.015
:4@H5-:3@H2'	3.54	3.24	3.78	5.12	5.11	5.115	0.005
:4@H5-:3@H3'	3.37	3.09	3.58	2.97	2.96	2.965	0.005
:4@H6-:3@H2'	2.79	2.58	2.95	3.34	3.35	3.345	0.005
:4@H6-:3@H3'	2.76	2.55	2.92	2.27	2.27	2.27	0
:4@H6-:4@H2'	2.88	2.66	3.05	2.95	2.88	2.915	0.035
:4@H6-:4@H3'	2.61	2.41	2.75	2.43	2.43	2.43	0
:4@H6-:4@H4'	3.96	3.58	4.28	4.09	4.09	4.09	0
:4@H6-:4@H5''	3.98	3.6	4.31	4.06	4.06	4.06	0

Supporting Figure 4: Overlap of representative structure for most populated clusters in Charmm36 277 K ensembles of GACC and CCCC tetranucleotides.

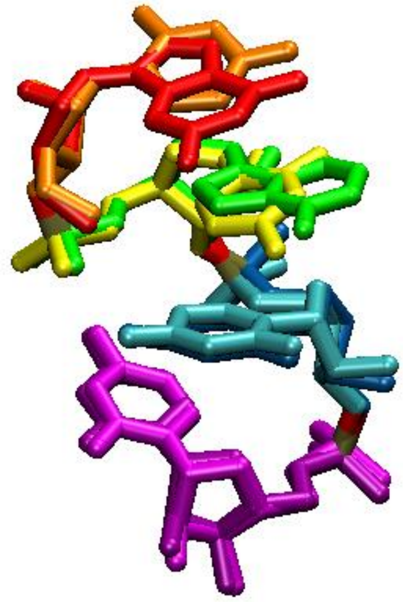

Supporting Figure 5: C36 backbone dihedral angle distributions for all structures in the top cluster for GACC and CCCC tetranucleotide.

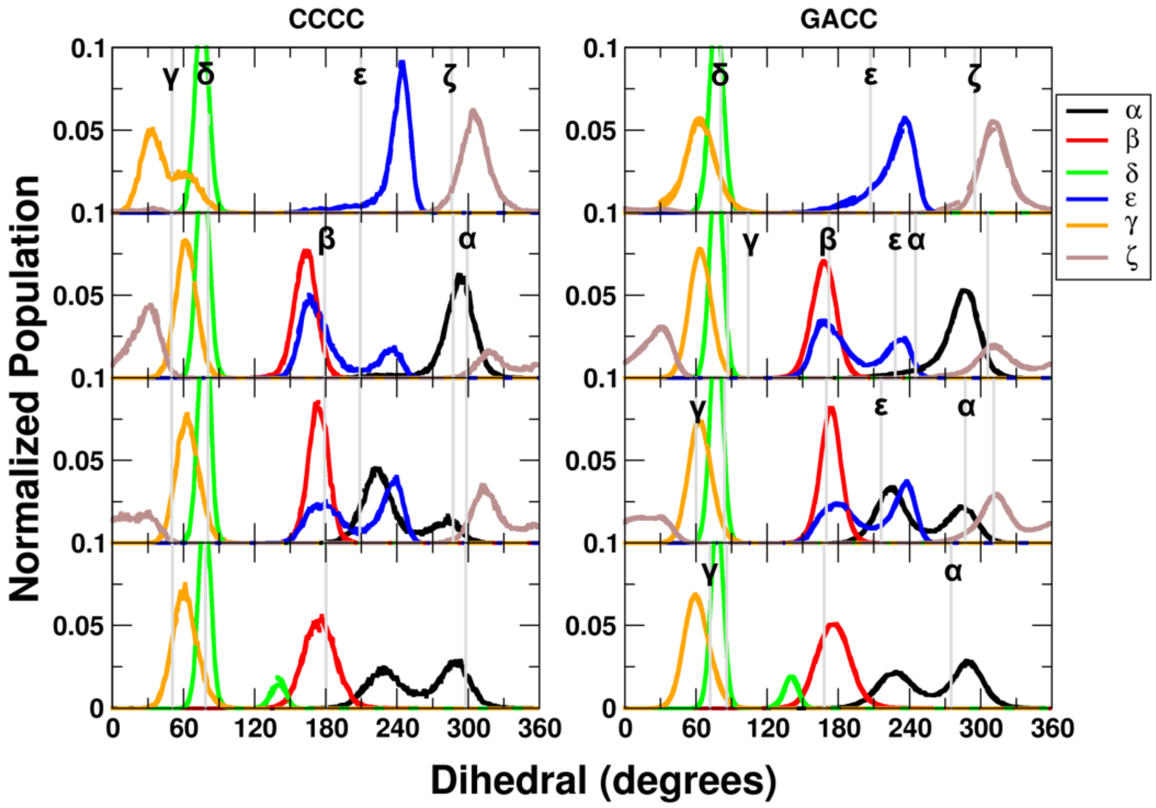

Values reported in the literature for each base are denoted by grey lines and labeled α-ζ. These are the expected values for the ensembles. The histogram peaks denote the actual dihedral angles adopted in the top cluster for C36. Though hard to see, two runs per tetranucleotide are shown as solid and dashed lines – this is perhaps most noticeable in GACC residue G1, zeta backbone dihedral.

Supporting Table 4: Hbond analysis for ff12 intercalated cluster trajectories.

Equivalent hydrogen bonds are aligned between CCCC (left) and GACC (right). Italicized red hydrogen bonds are less than 1% occupied, but were included if occupied at > 1% in the other system.

CCCC		Run 1 ff12, cluster 0, >1% occupancy			GACC		Run 1 ff12, cluster 2, >1% occupancy		
**Acceptor**	**DonorH**	**Fraction**	**AvgDist**	**AvgAng**	**Acceptor**	**DonorH**	**Fraction**	**AvgDist**	**AvgAng**
C_3@OP2	C5_1@HO5'	0.8948	2.7052	159.9835	C_3@OP2	G5_1@HO5'	0.8924	2.7325	161.7373
C_3@OP2	C3_4@HO3'	0.688	2.6288	164.8843	C_3@OP2	C3_4@HO3'	0.7251	2.6845	162.4274
C_3@OP1	C_2@HO2'	0.6786	2.7585	159.0779	C_3@OP1	A_2@HO2'	0.5273	2.754	160.1985
C3_4@OP1	C5_1@H41	0.4869	2.8858	165.0588	---	---	---	---	---
C3_4@OP1	C5_1@H42	0.4751	2.8884	165.0921	---	---	---	---	---
C_2@OP1	C_3@H41	0.4472	2.9377	164.5414	A_2@OP1	C_3@H41	0.3711	2.9445	165.8312
C_2@OP1	C_3@H42	0.3598	2.9365	164.5656	A_2@OP1	C_3@H42	0.3954	2.9416	165.7916
C_3@OP1	C3_4@HO3'	0.1915	2.6601	163.6221	C_3@OP1	C3_4@HO3'	0.1124	2.7201	161.9106
C_3@OP2	C3_4@HO2'	0.1613	2.6928	165.3785	C_3@OP2	C3_4@HO2'	0.1373	2.7009	165.1634
C_2@OP1	C5_1@HO2'	0.1343	2.8188	147.9376	A_2@OP1	G5_1@HO2'	0.1275	2.7804	150.7606
C_2@O3'	C5_1@HO5'	0.0592	3.1777	143.7227	A_2@O3'	G5_1@HO5'	0.0792	3.1254	145.1417
C3_4@O2	C5_1@HO5'	0.0252	2.9421	153.4401	C3_4@O2	G5_1@HO5'	0.0104	2.8812	155.5557
C3_4@OP1	C_3@HO2'	0.0137	2.9407	142.3319	C3_4@OP1	C_3@HO2'	0.5907	2.797	149.2362
C3_4@O2'	C5_1@HO5'	0.0133	2.9282	149.2251	*C3_4@O2'*	*G5_1@HO5'*	*0.0083*	*2.8955*	*150.9033*
C_3@OP1	C3_4@HO2'	0.0101	3.0447	151.123	*C_3@OP1*	*C3_4@HO2'*	*0.0026*	*3.2373*	*144.2653*
C5_1@O5'	*C3_4@HO3'*	*0.0074*	*3.2481*	*142.8123*	G5_1@O5'	C3_4@HO3'	0.0524	3.015	151.099
C_3@O5'	*C3_4@HO3'*	*0.0059*	*3.385*	*140.0611*	C_3@O5'	C3_4@HO3'	0.0178	3.2063	147.185
C_3@O4'	*C_2@HO2'*	*0.0002*	*2.7271*	*156.539*	C_3@O4'	A_2@HO2'	0.033	2.9831	150.9279
C_3@O5'	*C_2@HO2'*	*0.0001*	*3.2604*	*135.0656*	C_3@O5'	A_2@HO2'	0.0365	3.139	151.5214
C_3@O5'	*C5_1@HO5'*	*0.0044*	*3.2595*	*140.7711*	C_3@O5'	G5_1@HO5'	0.0792	3.0184	150.7065
C5_1@O5'	*C3_4@HO2'*	*0.0041*	*2.9793*	*152.0523*	G5_1@O5'	C3_4@HO2'	0.0584	2.9354	157.6017

Supporting Figure 6: Overlap of intercalated representative structures from GACC and CCCC.

RMSD of GACC to CCCC is 0.36 Å (mass weighted backbone heavy atoms only).

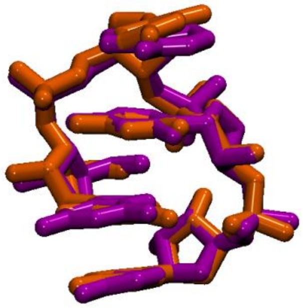

Supporting Figure 7a: Cluster correlation analysis for UUCG M-REMD simulations.

Last 1 μs of each 277 K and 300 K replica are used. Two independent M-REMD simulations were performed for each force field and their cluster populations show convergence between runs. Top: AMBER ff12; Bottom: AMBER ff12 + vdW_{all}.

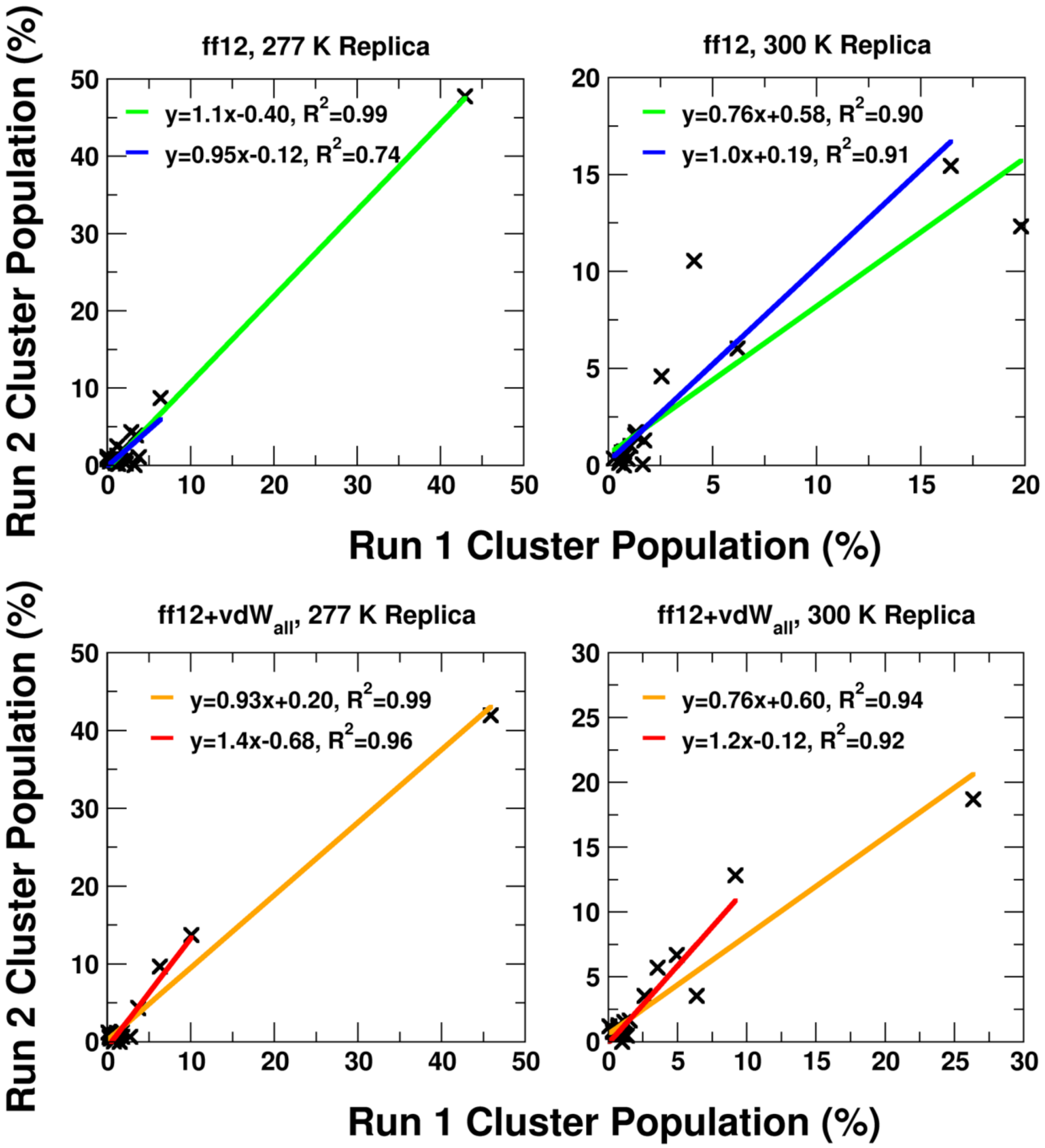

Supporting Figure 7b: Cluster correlation analysis for UUCG M-REMD simulations.

Last 1 μs of each 277 K and 300 K replica are used. Two independent M-REMD simulations were performed for each force field and their cluster populations show convergence between runs. Top: AMBER ff12 + vdW_{bb}; Bottom: AMBER ff99 + Chen-Garcia.

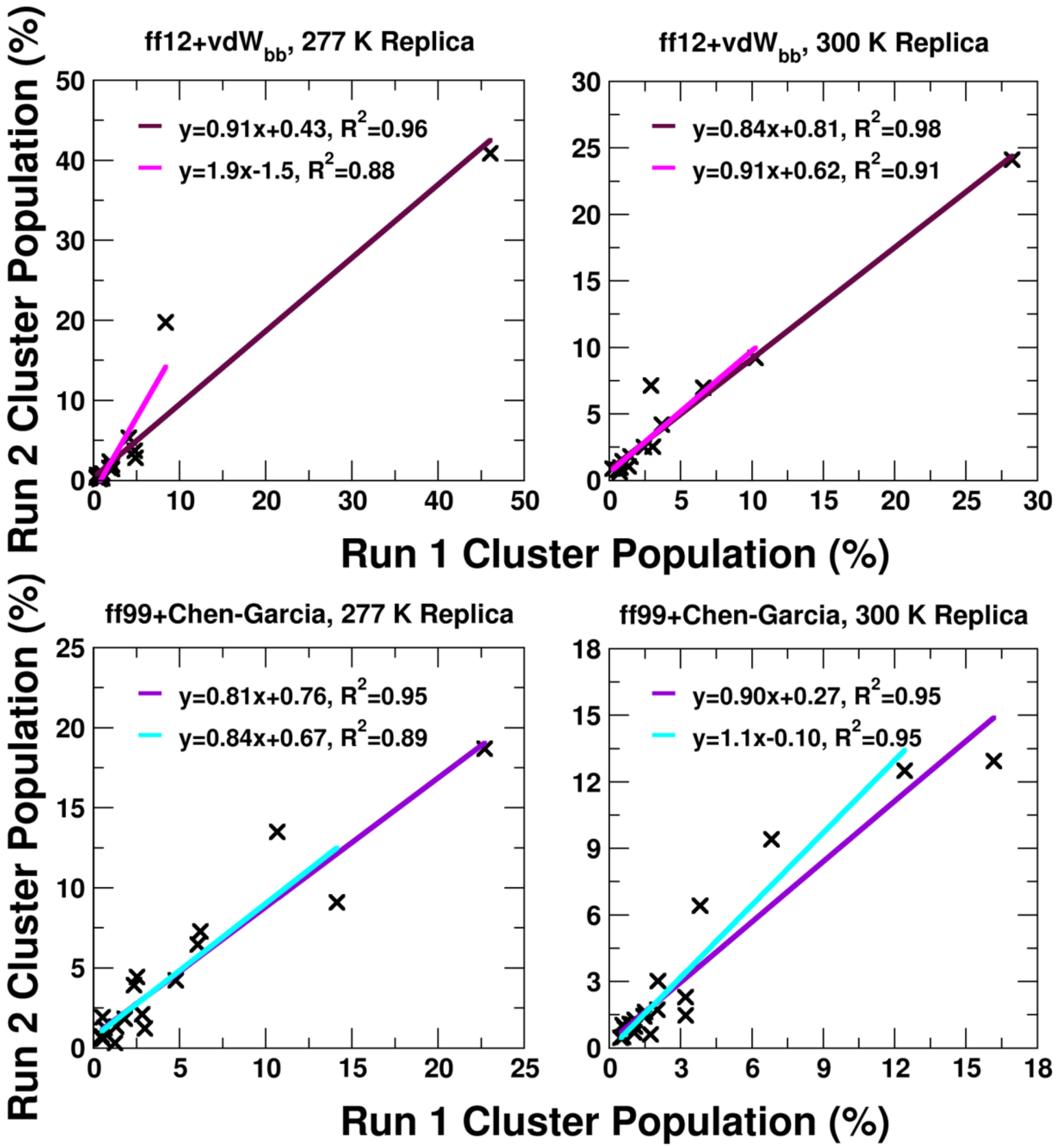

Supporting Figure 8: Close up of Cluster 2 from combined cluster analysis at 277 K coordinating a K+ ion.

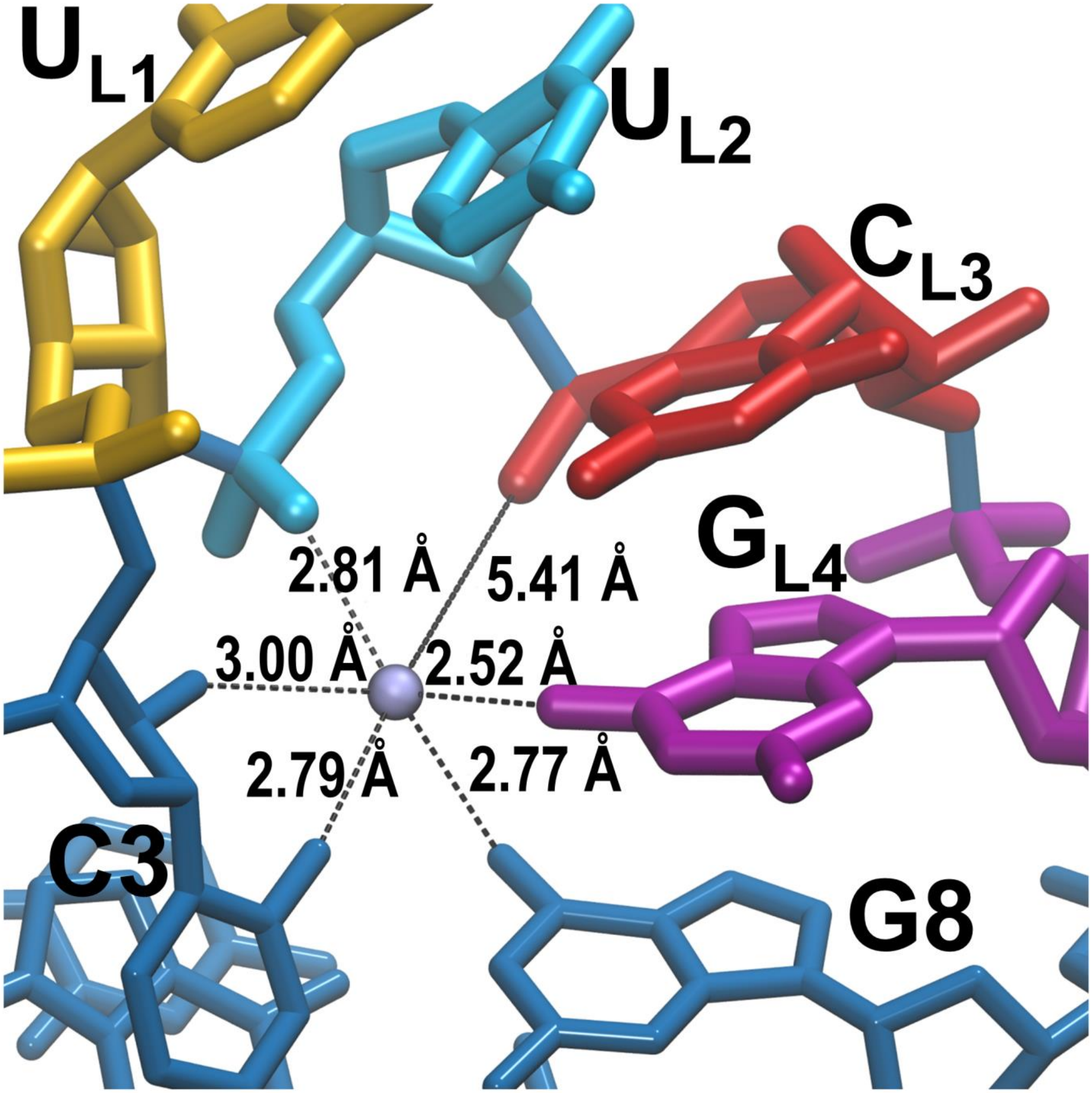

Supporting Figure 9: RMSD to ladder reference.

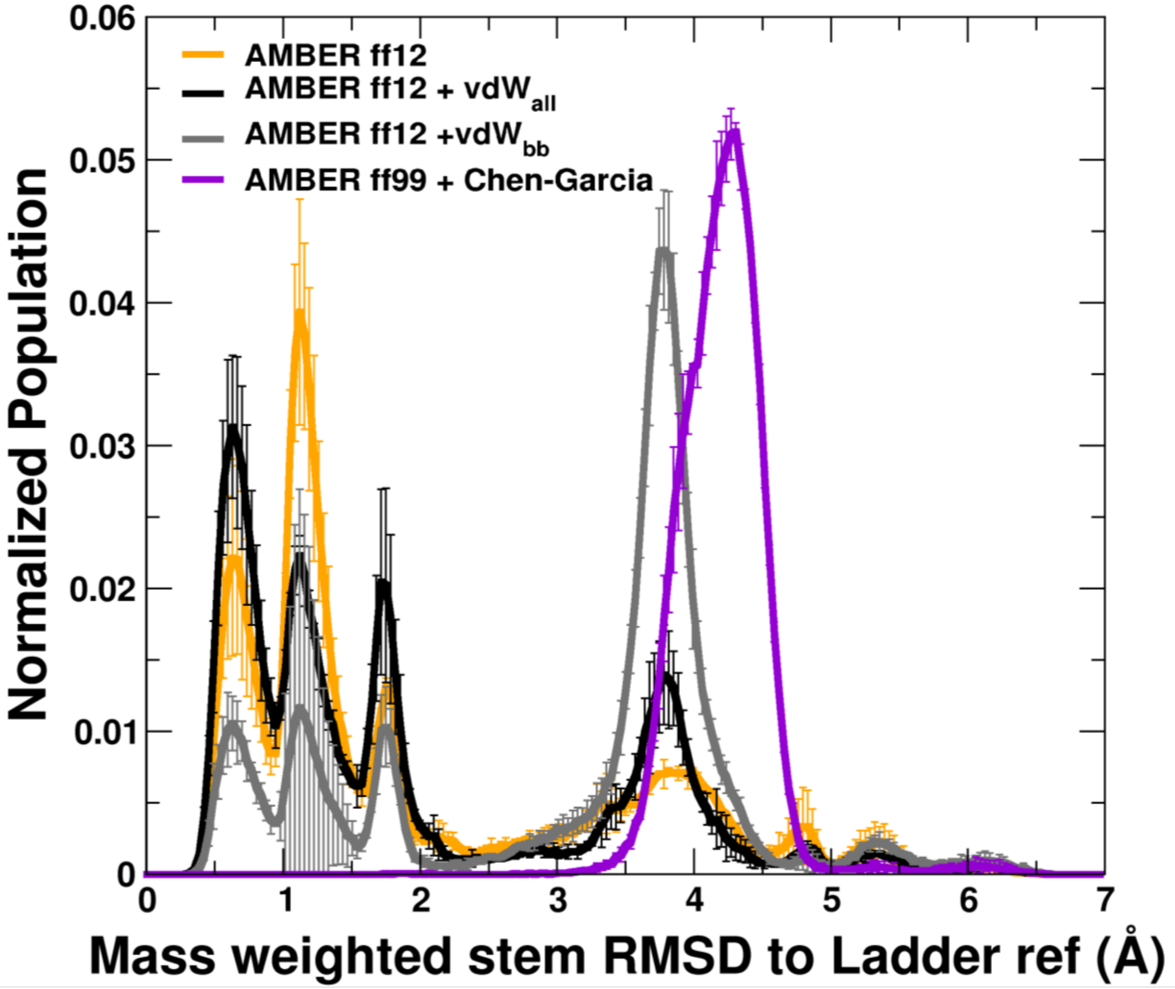

Average RMSD to a ladder-like stem reference structure of the last 1 μs of 277 K replicas for AMBER ff12, AMBER ff12 + vdW_{all}, AMBER ff12 + vdW_{bb}, and ff99 + Chen-Garcia systems. Reference structure is top cluster of 277 K combined cluster analysis, shown in main text Figure 4. Error bars represent standard deviation between two independent runs.

Supporting Figure 10: Unrestrained MD simulations starting from ladder representative structure do not transition back to a helix in 500 ns.

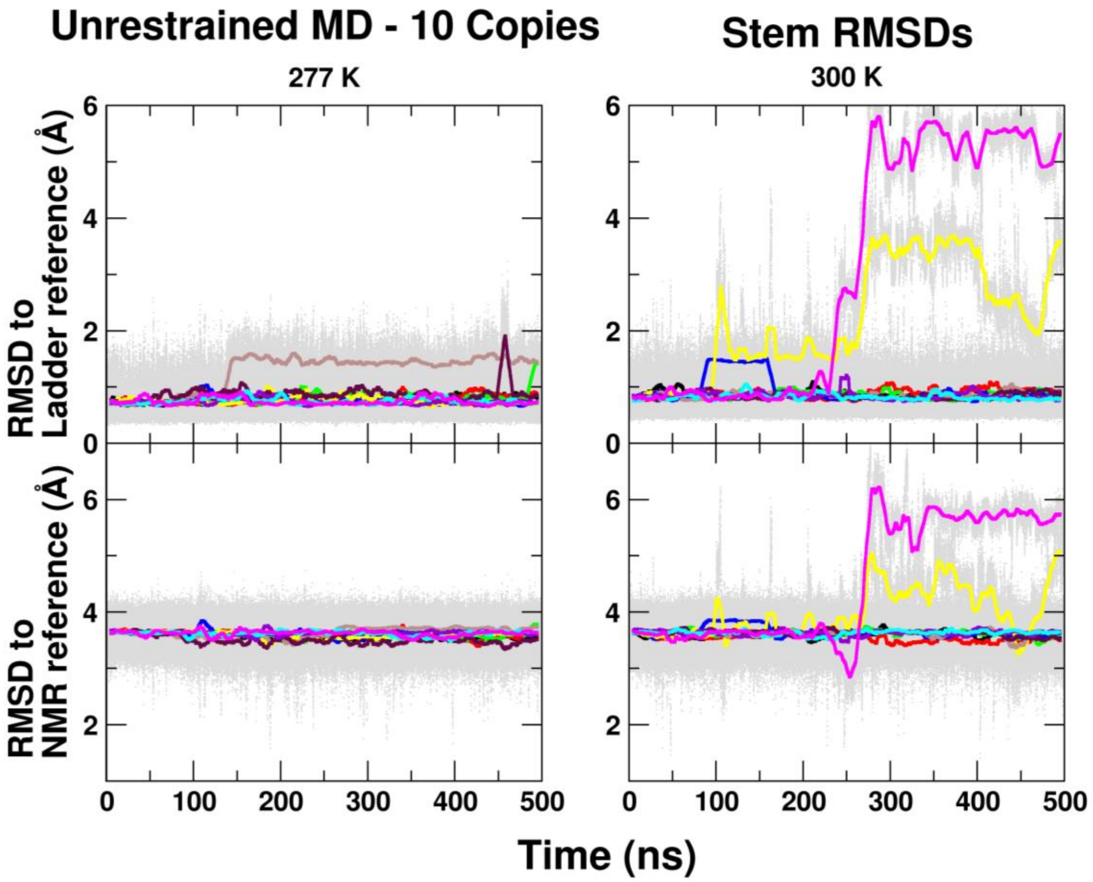

Top row: RMSD of the stem (:1-3,8-10) to the ladder reference structure at 277 K (left) and at 300 K (right). Bottom Row: RMSD of the stem to the NMR reference structure at 277 K (left) and at 300 K (right). It is important to note that two of the 300 K simulations move away from the ladder-like reference, but they do not move towards the NMR reference.

Supporting Figure 11: Combined clustering results for UUCG tetraloop simulations at 300 K.

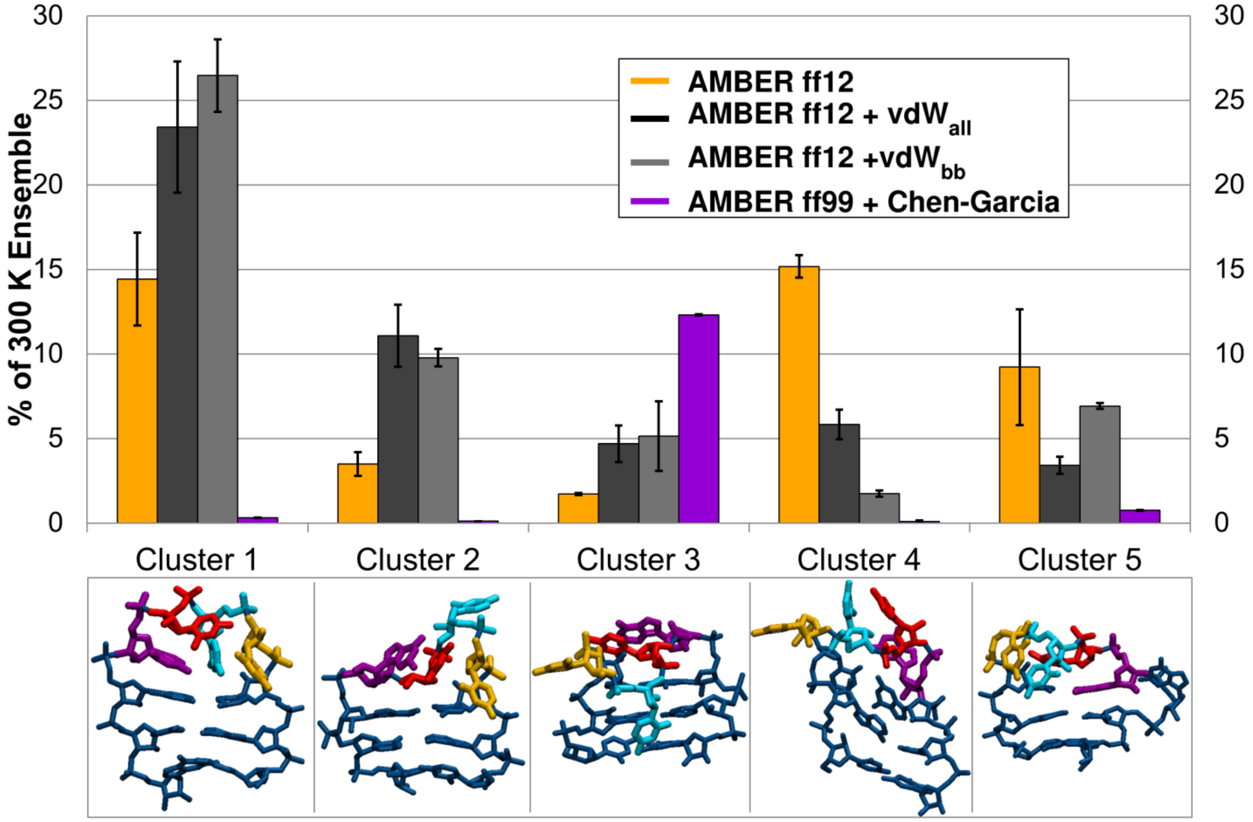

Cluster number is shown on the x-axis and percentage of each simulation's 300 K ensemble is shown on the y-axis. Representative structures are shown below the bar graph. The native structure is found in the 31st cluster, accounting for 0.02% of structures in the ff12 ensemble, 0.025% of structures in the ff12 + vdW_{all} ensemble, 0.07% of structures in the ff12 + vdW_{bb} ensemble and 0.97% of structures in ff99 + Chen-Garcia.

Supporting Figure 12: Cluster population vs. Time and cluster population correlation of unrestrained M-REMD UUCG folding simulations.

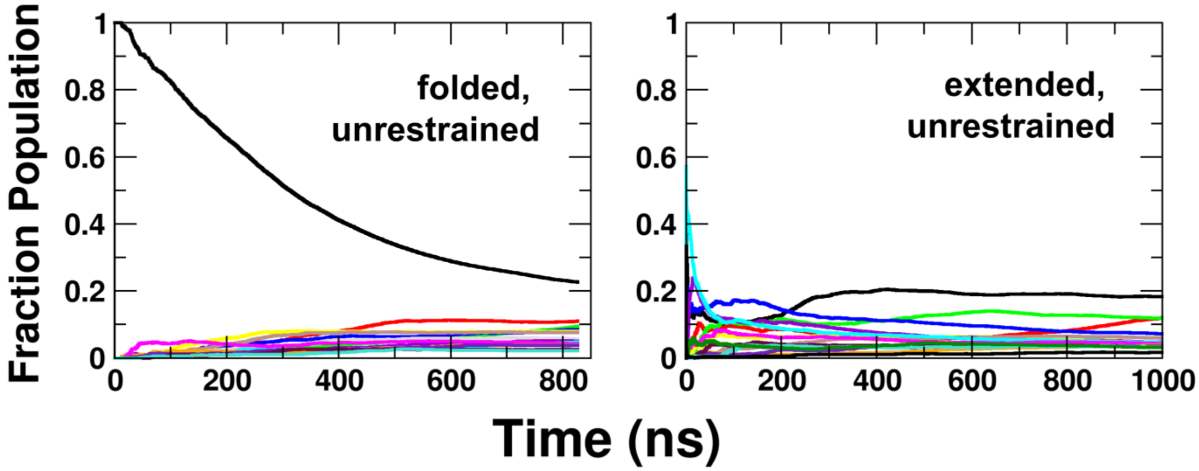

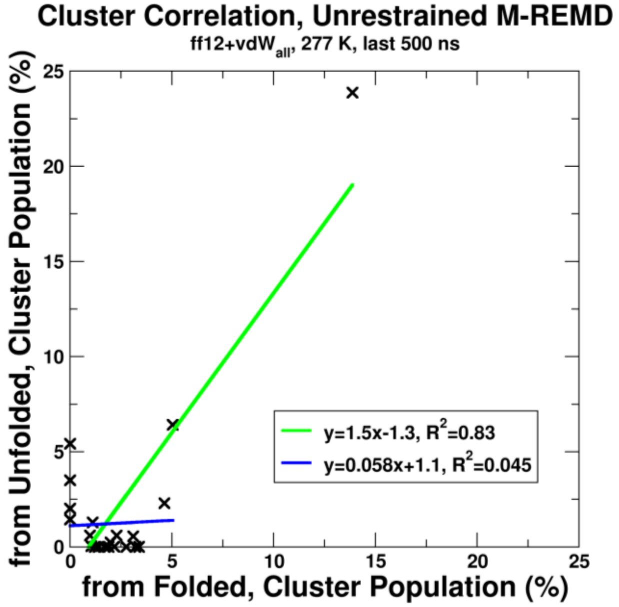

Top: Cluster population vs. Time of unrestrained M-REMD UUCG folding simulations from folded and unfolded starting structures. Bottom: Cluster Population correlation between the last 500 ns of unrestrained M-REMD simulations from folded and unfolded do not sample similar structure populations and remain unconverged.

Supporting Text: Anton Simulation Details

Production dynamics were performed on Anton using versions 2.4.1 and 2.4.5. Conversion of the Amber parameter and topology files into Anton appropriate formats was enabled by the supplied “amber_topNrst2cms.py” script on the computer anton.psc.edu with Desmond (Bowers et al. 2006) to create the needed *.cms file. A bug in the “amber_topNrst2cms.py” script erroneously assigned zero mass to C5’ atoms when converting from Amber topologies, so the files generated were hand-edited to fix the mass, and further checked to make sure the resulting *.cms file contained the correct Amber ff99 + parmbsc0 force field parameters. The Anton “guess_chem”, “refinesigma”, and “subboxer” programs were used to set up inputs for Anton and a series of “anton_run” commands performed to do the MD simulations. For the Anton runs, constant 300 K temperature and 1 bar pressure with weak coupling using a coupling time “tau” of 10.0, a maximum and minimum velocity scaling of 1.2 and 0.85, and a maximum and minimum expansion per step of 1.1 and 0.95 and kappa of 4.5x10-5 were imposed. The integration time step used was set to 2 fs and “max_strain” was set to 0.15 performing RESPA on the long-range non-bonded interactions every third step.(Tuckerman et al. 1992; Procacci et al. 1996)

Bowers KJ, Chow E, Xu H, Dror RO, Eastwood MP, Gregersen BA, Klepeis JL, Kolossváry I, Moraes MA, Sacerdoti FD, et al. 2006. Scalable algorithms for molecular dynamics simulations on commodity clusters. In *Proceedings of the ACM/IEEE Conference on Supercomputing*, pp. 11–17, Tampa.

Procacci P, Darden T, Marchi M. 1996. A very fast molecular dynamics method to simulate biomolecular systems with realistic electrostatic interactions. *J Phys Chem* **100**: 10464–10468.

Tuckerman M, Berne BJ, Martyna GJ. 1992. Reversible multiple time scale molecular dynamics. *J Chem Phys* **97**: 1990.

Supporting Table 5: aMD parameters used in M-REMD simulations of GACC with ff99 + χ_{Yil}.

Hamiltonian	Ethreshold (kcal/mol)	α (kcal/mol)
1	No Boost	No Boost
2	112.0	50.0
3	116.0	20.0
4	120.0	10.0
5	124.0	5.0
6	128.0	2.5
7	132.0	1.25
8	136.0	1.0

Supporting CPPTRAJ Script 1: Clustering command for analysis of GACC tetranucleotide simulations.

```
parm ../../nowat.GACC.parm7
trajin ../nowat.nc.0
#Set sieve so there are 10,000 frames during 1st pass
cluster dbscan minpoints 25 epsilon 0.9 \
  rms :1@N2,O6,C1',P,:2@H2,N6,C1',P,:3@O2,H5,C1',P,:4@O2,H5,C1',P \
  sievetoframe sieve 3 out cvt.dat summary summary.dat \
  repout rep repfmt pdb cpopvtime cpop.agr normframe
```

Supporting CPPTRAJ Script 2: Clustering command for analysis of CCCC tetranucleotide simulations.

```
parm ../nowat.cccc.topo
trajin ../nowat.277K.cccc.nc
cluster dbscan minpoints 25 epsilon 0.5 \
  rms :1@O2,H5,C1',P,:2@O2,H5,C1',P,:3@O2,H5,C1',P,:4@O2,H5,C1',P \
  sievetoframe sieve 10 out cvt.0.5.dat summary summary.0.5.dat \
  clusterout cluster.nc clusterfmt cdf repout rep.new repfmt pdb \
  cpopvtime cpop.0.5.agr normframe
```

Supporting CPPTRAJ Script 3. UUCG Combined Cluster Analysis

```
parm ../nowat.uucgREST.parm7
trajin ../trn-REST/2us/nowat.2us.300K.nc 100145 200144
trajin ../HMR-rest1/2us/nowat.2us.300K.nc 104001 204000
trajin ../ff12-run1/nowat.2.5us.300K.nc 145487 245486
trajin ../ff12-run2/nowat.2.5us.300K.nc 152894 252893
trajin ../ff99-chengar-run1/2us/nowat.cg.2us.300K.nc 98782 198781
trajin ../ff99-chengar-run2/nowat.2us.300K.nc 100001 200000
trajin ../ff12dacbb-run1/nowat.300K.nc 98223 198222
trajin ../ ff12dacbb-run2/nowat.300K.2us.nc 100001 200000

cluster dbscan minpoints 25 epsilon 1.5 rms mass :1-10 sievetoframe \
  sieve 60 summaryhalf split300.dat \
  splitframe 100000,200000,300000,400000,500000 \
  singlerepout singlerep300.mdcrd singlerepfmt crd \
  summary Replica0.summary.300.dat out cvt.300.dat
```

Supporting File: Frcmod.ff99CG – force modification file for building ff99 + Chen-Garcia LJ and dihedral modifications.

```
glycosdic bond torsion modifications to parm99 from Chen and Garcia (PNAS, 
2013, 110, 16820)
MASS
CA 12.01         0.360
CB 12.01         0.360
CK 12.01         0.360
CM 12.01         0.360
CQ 12.01         0.360
CP 12.01         0.360
CS 12.01         0.360
N* 14.01         0.530
NA 14.01         0.530
N2 14.01         0.530
NC 14.01         0.530
NB 14.01         0.530
O  16.00         0.434


DIHE
OS-CT-N*-CK    1       0.760994   50.0          1.0
OS-CT-N*-CM    1       0.760994   50.0         -1.0
OS-CT-N*-CM    1       0.505314   74.0          2.0

NONB
CA 1.812596614 0.068800
CB 1.812596614 0.068800
CK 1.812596614 0.068800
CM 1.812596614 0.068800
CQ 1.812596614 0.068800
CP 1.812596614 0.068800
CS 1.812596614 0.068800
N* 1.732800787 0.136002
NA 1.732800787 0.136002
N2 1.732800787 0.136002
NC 1.732800787 0.136002
NB 1.732800787 0.136002
O  1.634259844 0.168000
```

Supporting File: Parmed.py script for modifying off-diagonal LJ terms for ff99 + Chen-Garcia.

```
printLJTypes @%CA
printLJTypes @%NA
printLJTypes @%O
changeLJPair @%CA @%OW 3.67622 0.102933
changeLJPair @%NA @%OW 3.592215 0.144722
changeLJPair @%O  @%OW 3.429413 0.160848
parmout ff99chengarcia.parm7
go
```

Supporting File: Frcmod.vdW – force modification file for building ff12 + vdW_{all} modifications.

```
Steinbrecher-Latzer-Case Phosphate Oxygen parameters (JCTC, 8, 4405-12, 2012)

MASS
   O2 16.00         0.434
   OS 16.00         0.465

NONB
   O2           1.7493  0.2100
   OS           1.7718  0.1700
```

Supporting File: Parmed.py script for modifying O4' atom from ff12 + vdW_{all} to default ff12, generating ff12 + vdW_{bb} modifications.

```
addLJType @O4' radius 1.6837 epsilon 0.1700
parmout ff12vdWbb.parm7
go
```
